# In silico design of the multi-epitope vaccine for lung adenocarcinoma based on hub gene-derived neoantigens

**DOI:** 10.1186/s12885-026-15765-1

**Published:** 2026-03-06

**Authors:** Shaoshuai Li, Minjia Huang, Liqiu Jia, Siyu Liu, Xueting Sun, Daxia Cai, Yanping Su, Minjiang Chen, Chenying Lu, Jiansheng Huang, Jianfei Tu, Jiansong Ji

**Affiliations:** 1 International Clinical School, Hangzhou Medical College (Lishui Central Hospital), Lishui, Zhejiang 323000 China; 2 Department of Clinical Laboratory, Lishui Central Hospital, The Fifth Affiliated Hospital of Wenzhou Medical University, Lishui, Zhejiang 323000 China; 3 Zhejiang Key Laboratory of Imaging and Interventional Medicine, Zhejiang Engineering Research Center of Interventional Medicine Engineering and Biotechnology, Lishui Central Hospital, The Fifth Affiliated Hospital of Wenzhou Medical University, Lishui, Zhejiang 323000 China; 4 Department of Radiology, Lishui Central Hospital, The Fifth Affiliated Hospital of Wenzhou Medical University, Lishui, Zhejiang 323000 China; 5 Department of Cancer Center, Lishui Central Hospital, The Fifth Affiliated Hospital of Wenzhou Medical University, Lishui, Zhejiang 323000 China; 6 Key Laboratory of Comprehensive Diagnosis and Treatment for Thoracic and Abdominal Tumors, Cancer Center, Lishui Central Hospital, The Fifth Affiliated Hospital of Wenzhou Medical University, Lishui, Zhejiang 323000 China

**Keywords:** Neoantigen, Multi-epitope vaccine, Hub gene, Immunoinformatics, Reverse vaccinology, In silico

## Abstract

**Background:**

Lung adenocarcinoma (LUAD) is the most prevalent and lethal subtype of non-small cell lung cancer (NSCLC), characterized by an unfavorable 5-year survival rate ranging from 10% to 20%. Neoantigen-based vaccine platforms show encouraging benefits for NSCLC patients. However, the vaccine efficacy may be limited, partially due to low immunogenicity and an immunosuppressive tumor microenvironment. This study aims to design a multiple epitopes vaccine targeting neoantigens derived from hub genes in LUAD, using immunoinformatics based strategies to explore a potential immunotherapeutic approach for LUAD.

**Method:**

Multiple GEO datasets were ultilized to identify the up-regulated genes in LUAD. Protein-protein interaction networks were analyzed and hub gene were identified based on overlapping top-ranked nodes across five topological algorithms in cytoscape. Hub gene derived neoantigens were identified using TSNAdb v2.0 and futher screened for LUAD in the cBioportal database. Neoantigen-derived CTL epitopes were predicted across all 12 MHC class I supertypes and subsequently screened for antigenicity, allergenicity, and toxicity. Linear B-cell epitopes were predicted from the extracellular region of PD-L1 using established B-cell epitope prediction methods. The multi-epitope vaccine (MEV) was constructed by rationally conjugating selected CTL and B cell epitopes using appropriate peptide linker.

**Results:**

114 differential express genes were identified from GEO dataset in LUAD. Subsequently, ten hub genes were identified and validated and their expression was associated with poorer overall survival in patients with LUAD. Tumor specific neoantigens were screened from these hub genes, and eight neoantigen epitopes with antigencity, non-allergenicity and non-toxicity were selected as cytotoxic T lymphocyte (CTL) epitopes for multi-epitopes vaccine construction. The vaccine was further incorporated predicted PD-L1 derived linear B-cell epitopes, pan HLA DR-binding epitope (PADRE), a universal helper T-cell epitope and β-defensin to augment protective efficacy. The designed and optimized vaccine possessed properties of solubility, antigenicity, non-allergenicity, and non-toxicity. Molecular docking demonstrated stable and favorable binding interactions between MEV and TLR2, TLR3 and TLR4 complex, as validated by molecular dynamics simulations. Immune simulation analysis revealed that MEV had the potential to elicit a series of T cell and B cell specific immune responses. Finally, the optimized MEV was cloned in silico and successfully expressed in eukaryotic cells.

**Conclusions:**

Our findings suggest that hub genes may serve as a promising source of neoantigens in lung adenocarcinoma. The multiple epitopes vaccine engineered from these hub genes shows potential for stimulating immune responses which highlights the potential of hub genes as prioritized candidates for advancing neoantigen-based vaccine development against LUAD.

**Supplementary Information:**

The online version contains supplementary material available at 10.1186/s12885-026-15765-1.

## Background

Lung cancer remains the leading causes of cancer-related death worldwide, with an estimated 2.2 million new cases and 1.8 million deaths in 2020 [1]. Non-small-cell lung cancer (NSCLC) accounts for approximately 85% of lung cancer cases. Lung adenocarcinoma (LUAD) is the most common and pathological type of NSCLC. The treatment of LUAD has evolved from traditional approaches, such as surgery, radiotherapy, and chemotherapy, to more advanced strategies that include molecular targeting and immunotherapy [2, 3]. Immune checkpoint inhibitors (ICIs)-based immunotherapy has become the standard choice for advanced NSCLC patients, as it can restore impaired T-cell activity and effectively attack tumor cells [4, 5]. However, ICIs-based immunotherapy has several limitations, such as low response rates and severe side effects that can be partly attributed to the reduction of infiltrating T cells, especially tumor specific T cells [6]. Hence, new strategies are urgently needed to further augment the infiltration of tumor-specific T cells and stimulate the immune response.

Cancer vaccines, comprising tumor lysates, viral vector vaccines, cell-based vaccines, gene-based vaccines (DNA or RNA), and peptide-based vaccines, are regarded as promising and effective for stimulating strong and long-lasting host immune responses [7–11]. Among these cancer vaccine platforms, multi-epitope vaccines(MEVs) based on bioinformatics have the following unique properties [12]: (I) they consist of B-cell, T-helper (Th) cell and/or cytotoxic T-lymphocyte (CTL) epitopes that can induce both cellular and humoral immunity precisely and concurrently; (II) they comprise several antigen epitopes that cover a wide range of target antigens; and (III) they are devoid undesirable antigen components, thus preventing damaging immune responses and adverse side effects. Previous preclinical studies have shown promising results with MEVs, including enhanced Th and CTL responses and increased IFN-γ production in several solid tumors [13, 14]. During the design of MEVs, selecting the appropriate tumor antigen is a crucial step for ensuring vaccine effectiveness. These antigens can be categorized into two main types: tumor-associated antigens (TAAs) and tumor-specific antigens (TSAs). TAAs are self-antigens that are abnormally expressed or over-expressed in tumors, for example, melanoma antigen family A, 3 (MAGE-A3) [15], mucin 1 [16] and New York esophageal squamous cell carcinoma 1(NY-ESO-1) [17]. Previous studies have shown that the clinical efficacy of vaccines targeting these TAAs is limited [18, 19], probably because TAA specific T cells are subject to central or peripheral tolerance [20]. In contrast, TSAs generated by tumor specific mutations are non-self-antigens that can evade immune tolerance and display a higher affinity for MHC molecules and T cell receptors [21]. Thus, neoantigens are considered valuable targets for cancer vaccines. With the development of powerful sequencing techniques, such as next-generation sequencing, RNA sequencing, and whole exome sequencing, potential neoantigens have been identified on a large-scale, and the corresponding immunogenic epitopes have been predicted by bioinformatics [22]. In recent years, personalized neoantigen (exclusive to individual patients) vaccines have shown clinical benefits in various cancers, but the associated time and costs remain challenges [23–25]. To address these limitations, high frequency mutations in various cancers, such as KRAS, ALK and EGFR, have been used as shared neoantigens to construct epitope vaccines [26–28]. However, new strategies for selecting suitable neoantigens that can trigger genuine anti-tumor immune responses must be explored.

Hub genes are highly connected within biological networks and play a central role in various biological pathways and cellular functions [29]. Hub nodes, with a high connection degree and betweenness centrality, have been proposed as promising drug targets for effectively suppressing tumor growth, due to their inherent ability to interact with other proteins [29, 30]. In recent years, several hub genes, such as CDC20, TOP2A, AURKA, and MELK, have been extensively discussed in lung adenocarcinoma based on their central regulatory roles in tumor progression, prognosis, or therapeutic efficacy. These genes have been summarized in multiple reviews as potential molecular targets across solid tumors, including lung adenocarcinoma [31–34]. In addition, multiple integrative bioinformatics studies have identified them as hub genes in LUAD through network-based analyses [35–37]. However, these hub genes have not been systematically explored as sources of tumor specific neoantigens for multiepitope vaccine design. Currently, despite advances in cancer vaccines, including peptide, cell, DNA, and mRNA-based platforms, antigen selection remains a critical challenge. Peptide vaccines often target a limited number of epitopes, while cell-based vaccines may include self-antigens, increasing the risk of side effects. Identifying immunodominant antigens and designing vaccines that balance immune activation with low toxicity remains a major barrier. By targeting hub genes that occupy central positions in tumor associated networks, a hub gene derived MEV strategy may offer a rational approach to select recurrent, functionally relevant neoantigens, thereby improving target selection and enhancing immune coverage.

**Fig. 1:**
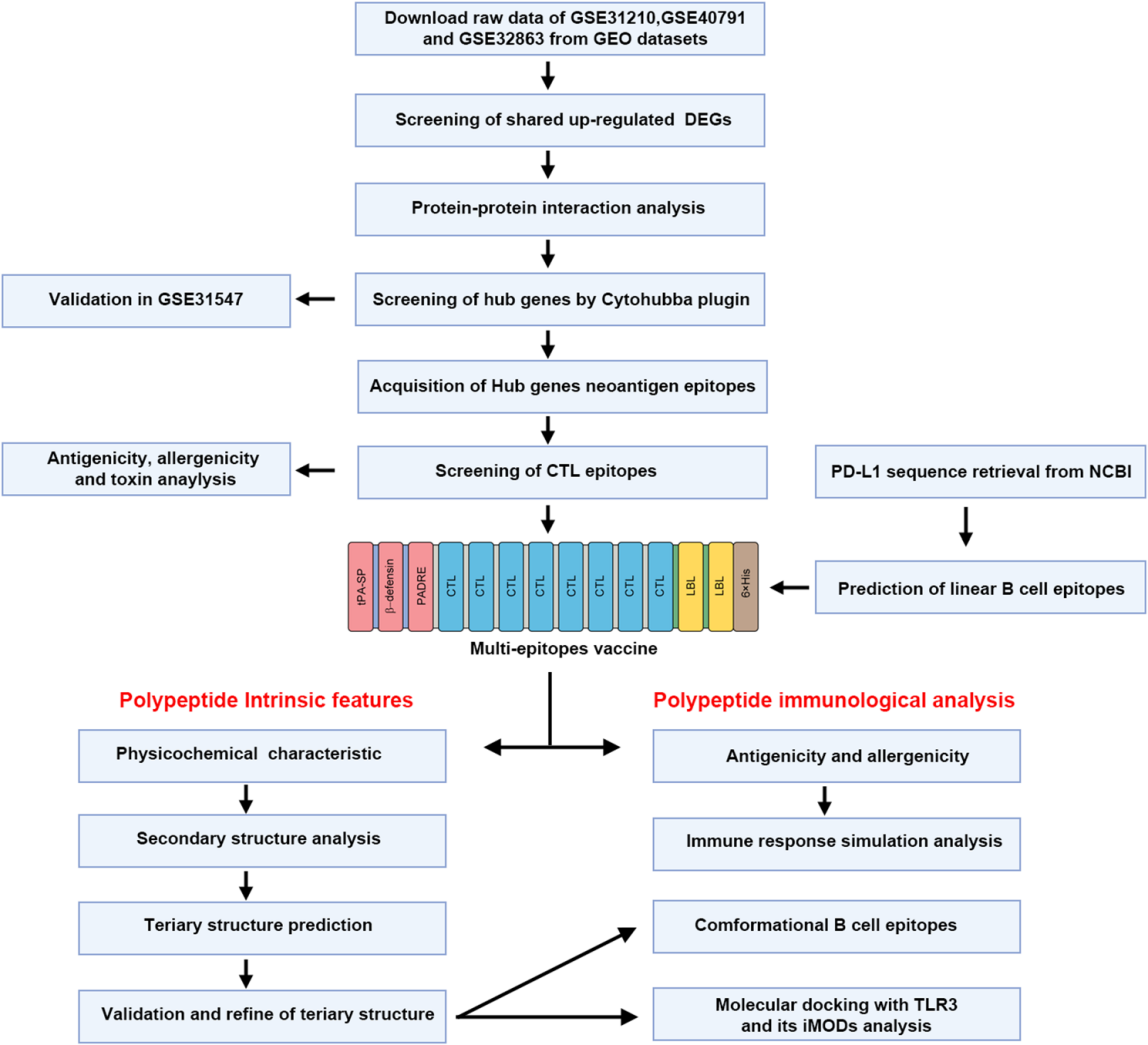
Overall workflow of multi-epitope vaccine design for lung adenocarcinoma

In this study, we proposed utilizing highly expressed hub genes to screen neoantigens. Subsequently, the epitopes derived from these neoantigens were identified through in silico analysis to design a multi-epitope vaccine. Highly upregulated hub genes in human LUAD were screened using multiple GEO databases. Tumor neoantigen peptides and corresponding CTL epitopes were predicted by using immunoinformatics tools. CTL epitopes with antigenicity, non-allergenicity, and non-toxicity were selected to construct the vaccine. Additionally, Linear B cell epitopes from PD-L1 were identified and introduced into the vaccine to restore impaired T cell function and further enhance the T-cell-mediated anti-tumor immune response. Furthermore, Vaccine immunogenicity was improved by introducing the universal helper T cell epitope PADRE, a signal peptide, β-defensin, and corresponding linkers. In silico, we evaluated the physicochemical properties, antigenicity, and allergenicity of the vaccine and predicted its secondary and tertiary structures. Molecular docking and molecular dynamics simulations were conducted for interactions between the vaccine and Toll-like receptor 3 (TLR3). To further evaluate the immune responses induced by the vaccine, an online immune response simulation analysis was conducted. The overall flow diagram for the in silico design of an MEV against LUAD is presented in Fig. 1.

## Methods

### Data retrieval and DEG identification

Gene expression profiles (GSE31210, GSE40791, GSE32863, and GSE31547) derived from human tissue were downloaded from the Gene Expression Omnibus (GEO) database (http://www.ncbi.nlm.nih.gov/geo/). All selected GEO datasets were generated using microarray platforms, allowing direct cross cohort integration and consistent differential expression analysis using the limma framework. These datasets were selected to enable cross cohort validation and to identify robust transcriptional signatures consistently associated with LUAD across independent populations. GSE31210, GSE40791, and GSE32863 were used to screen differentially expressed genes (DEGs), while the GSE31547 dataset was used to validate the upregulated DEGs shared by the previous three datasets. The GSE31210 dataset comprises 226 LUAD and 20 normal lung tissue samples. The GSE40791 dataset includes 94 LUAD and 100 normal lung tissue samples. The GSE32863 dataset consists of 58 LUAD and 58 normal lung tissue samples. The GSE31547 dataset contains 30 LUAD and 20 normal lung tissue samples. Differential expression analysis was conducted on the GSE31210, GSE40791, and GSE32863 datasets by using the R limma package (Version 3.40.2). Upregulated DEGs in tumors were identified by using log |fold change| > 1 and an adjusted P-value < 0.05 as the threshold criteria. This threshold was selected as a commonly used and widely accepted cutoff to balance biological relevance and statistical robustness. The overlapping DEGs among the three datasets were screened and presented as a Venn diagram by using the online tool Venny 2.0 (https://www.bioinformatics.com.cn/static/others/jvenn/example.html). Integrative DEGs derived from multiple independent GEO datasets were used to define a robust tumor specific gene signature for hub gene identification. Similar integrative DEG screening and networ based gene prioritization strategies have been widely applied in previous cancer bioinformatics studies [38, 39].

### Hub gene identification and validation

Protein-protein interactions (PPIs) was performed on the overlapping upregulated DEGs shared by all three datasets using the online Search Tool for the Retrieval of Interacting Genes/Proteins (STRING, http://www.string-db.org). Only high confidence interaction pairs with a combined interaction score ≥ 0.9 were selected and subsequently, the result of PPI networks was visualized using the Cytoscape software (Version 3.9.1). To identify hub genes within the PPI networks, five ranking algorithms of the cytoHubba plugin in Cytoscape were utilized, including three local ranking algorithms (Maximal Clique Centrality [MCC], Maximum Neighborhood Component [MNC], and Degree) and two global ranking algorithms (Stress Centrality and Betweenness Centrality) [40]. For each algorithm, the top 15 ranked genes were selected based on their respective centrality scores. Hub genes were defined as those consistently ranked among the top candidates across five topology algorithms to ensure robustness and reduce method specific bias. The venny package was further utilized to identify 10 hub genes from the top 15 DEGs of each algorithm. Finally, the expression of the selected 10 hub genes was validated in LUAD and normal lung tissue based on the GSE31547 dataset.

### Kaplan-Meier analysis of hub genes

The prognostic values of the hub genes were estimated using an online Kaplan-Meier plotter (K-M plotter, http://www.kmplot.com). A total of 1161 LUAD samples were divided into two groups based on the expression level of hub genes. The 5-year survival rates of these lung cancer patients were presented as hazard ratios (HRs) along with 95% confidence intervals and log-rank P-values.

### Neoantigen prediction of hub genes

Tumor-specific neoantigen database version 2.0 (TSNAdb v2.0, https://pgx.zju.edu.cn/tsnadb/) is an online tool to identify single nucleotide variants (SNV), insertions and deletions (INDEL), or fusion-derived neoantigens with high confidence by combining the prediction results of DeepHLApan, MHCflurry, and NetMHCpan v4.0. Only the peptide-HLA complexes that met all the criteria of these tools were regarded as potential neoantigens. All hub gene neoantigens for lung cancer were identified using TSNAdb v2.0 and futher screened for LUAD in the cBioportal database.

### GO and KEGG enrichment analysis

Functional annotation of the differentially expressed genes (DEGs) and hub genes was conducted using the Enrichr online platform (https://maayanlab.cloud/Enrichr/), including Gene Ontology (GO) and Kyoto Encyclopedia of Genes and Genomes (KEGG) pathway enrichment analyses. Enrichment terms with an adjusted P values < 0.05 were considered statistically significant. The enrichment results were subsequently visualized using the BMKCloud platform (www.biocloud.net).

### B cell epitope prediction

PD-L1 was selected as the B cell antigen due to its extracellular domain is accessible to antibody recognition and has well established relevance in LUAD immunotherapy [41, 42]. Targeting extracellular PD-L1 epitopes was intended to induce a humoral immune component that complements CTL mediated antitumor immunity, rather than to replace existing checkpoint blockade strategies. The immune checkpoint PD-L1 protein sequence (ID: Q9NZQ7) was retrieved from the Uniprot database (http://www.uniprot.org). Linear B cell epitopes of PD-L1 extracellular sequence (residues 19 to 238) were predicted via the online server Immune Epitope Database (IEDB, http://www.iedb.org) with the method of Bepipred linear epitopes prediction 2.0. In addition, the BCpreds server (http://ailab.ist.psu.edu/bcpred/predict.html) was used with the settings of 14 amino acids length and 90% sensitivity. To improve the reliability of B cell epitope prediction and reduce tool specific bias, only epitopes shared by both servers were selected as PD-L1 epitopes for subsequent vaccine design. To assess potential off-target cross reactivity, the selected PD-L1 B cell epitopes were screened for sequence similarity against the human proteome using BLASTp optimized for short peptide queries.

### CTL epitope prediction

NetCTL v1.2 (http://www.cbs.dtu.dk/services/NetCTL/) was utilized to CTL epitopes of hub genes for all 12 MHC I supertypes (A1, A2, A3, A24, A26, B7, B8, B27, B39, B44, B58, and B62). The threshold value of 0.75 having a sensitivity of 0.80 and specificity of 0.97, was used for prediction. Subsequently, the selected epitopes were refined for antigenicity, allergenicity, and toxicity by using the online servers VaxiJen v2.0, AllerTOP v2.0, and ToxinPred respectively. The CTL epitopes having antigenicity, non-allergenicity, and non-toxicity were selected for further vaccine construction.

### Population coverage analysis

Population coverage analysis was performed using the IEDB Population Coverage Analysis tool (http://tools.iedb.org/population/) [43]. Predicted T-cell epitopes were combined with their corresponding MHC class I restrictions and used as input for the analysis. This tool was used to estimate the proportion of individuals with the potential to present at least one epitope across human populations.

### Multi-epitope vaccine design

The MEV was formulated by conjugating the selected CTL and B-cell epitopes. Briefly, the linear CTL epitopes were linked with an AAY linker, and the two linear B cell epitopes (HQVLSGKTTTTNSKREEK and FRRLDPEENHTAEL) were linked with a KK linker. The pan HLA DR-binding epitope (PADRE) that acts as a T helper epitope can facilitate the presentation of other epitopes to T cells [44]. Hence, the PADRE sequence (AKFVAAWTLKAAA) was conjugated to the first CTL epitope with an AAY linker. Additionally, to increase the immunogenicity of the vaccine, human β-defensin sequence (GIINTLQKYYCRVRGGRCAVLSCLPKEEQIGKCSTRGRKCCRRKK)was retrieved from the literature [45] and served as an adjuvant. β-defensin was selected as the adjuvant for this vaccine design based on its endogenous host defense peptide properties and well characterized immunomodulatory functions. This sequence was then conjugated to PADRE to augment the immune response. Furthermore, a tissue plasminogen activator signal peptide sequence (tPA-SP) was conjugated to the β-defensin to augment the expression and secretion of the protein [46]. The EAAAK linker was used to join signal peptide, β-defensin, and PADRE sequences. Finally, a poly-histidine tag (His-tag) was introduced to the C-terminal of the vaccine to examine protein expression.

### Prediction of antigenicity, allergenicity, solubility and physicochemical properties of the MEV

To analyze MEV antigenicity, two online servers, VaxiJen v2.0 (https://www.ddg-pharmfac.net/vaxijen/VaxiJen/VaxiJen.html) and ANTIGENpro (http://scratch.proteomics.ics.uci.edu/) were used. Simultaneously, MEV allergenicity was evaluated via the online servers AllerTOP v2.0 (https://www.ddg-pharmfac.net/AllerTOP/) and AllergenFP v1.0 (http://ddg-pharmfac.net/AllergenFP/). Furthermore, the physicochemical properties of the MEV were analyzed via the ProtParam server (https://web.expasy.org/protparam/), including theoretical isoelectric point (PI), half-life, instability index, and aliphatic index [47]. Finally, the solubility of the MEV was predicted via the Protein-Sol server (https://protein-sol.manchester.ac.uk/), where a score above the threshold of 0.45 was considered to indicate good solubility.

### Secondary structure prediction

PSIPRED is a simple and accurate secondary structure prediction method and can effectively predict α-helices, β-sheets, and random coils [48]. Hence, the PSIPRED v3.3 server (http://bioinf.cs.ucl.ac.uk/psipred/) was utilized to predict the secondary structure of MEV. Subsequently, the RaptorX Property tool (http://raptorx6.uchicago.edu/StructurePropertyPred/predict/) was employed to predict the secondary structural properties of the MEV without using any templates.

### Tertiary structure prediction, refinement, and validation

The 3Dpro server (http://scratch.proteomics.ics.uci.edu/) is most appropriate to predict the structure of proteins without good structure templates [49]. Therefore, the tertiary structure of the MEV was predicted utilizing the 3Dpro server (http://scratch.proteomics.ics.uci.edu/). Subsequently, the GalaxyRefine online server (http://galaxy.seoklab.org/cgi-bin/submit.cgi? type=REFINE) was used to optimize the initial tertiary structure by refining side chains, repackaging them, and performing overall structural relaxation through molecular dynamics simulations. GalaxyRefine has several parameters, including global distance test-high accuracy (GDT-HA), root-mean-square deviation (RMSD), and the MolProbity score. The three components of MolProbity were also analyzed, including the Ramachandran favored score (Rama favored), the number of atomic clashes per 1000 atoms (Clash score), and the percentage of rotamer outliers (poor rotamers). The validation of tertiary structure before and after refinement was performed using the ProSA-web server (https://prosa.services.came.sbg.ac.at/prosa.php) with Z-score [50]. A Z-score of the tertiary structure of the MEV falling into the area of natural proteins indicates tertiary structural accuracy. Additionally, the ERRAT server (https://saves.mbi.ucla.edu/) was utilized to evaluate the validation of the tertiary structure by analyzing non-bonding atomic interactions. The overall quality factor generated by ERRAT analysis typically ranges from 0 to 100, with higher scores indicating that the structure is closer to that of a native protein. Lower scores suggest that the model may contain unreasonable regions or errors. Finally, a Ramachandran diagrams was created using the PDBsum Generate (https://www.ebi.ac.uk/thornton-srv/databases/pdbsum/Generate.html) to evaluate the quality of the modeled structure based on the percentage and number of residues in the most favored regions, additional allowed regions, generously allowed regions, and disallowed regions.

### Prediction of conformational B cell epitopes

More than 90% of B-cell epitopes of the protein were discontinuous epitopes. Therefore, the ElliPro online server (http://tools.iedb.org/ellipro/) was used to predict the discontinuous or conformational B-cell epitopes of the 3D model with default parameters.

### Molecular docking

Based on the immunological characteristics of the vaccine construct, Toll-like receptors involved in both nucleic acid and protein mediated innate immune sensing were selected for molecular docking analyses. TLR3 was included to represent nucleic acid sensing pathways potentially engaged during intracellular processing and expression of the DNA vaccine. In addition, given that the construct encodes human β-defensin-3 (hBD-3), a protein-based immunomodulatory component known to activate antigen presenting cells via the TLR1/2 axis [51], docking analyses were extended to the TLR1/2 heterodimer and the TLR4–MD-2 complex to evaluate potential defensin-associated pattern recognition. The three-dimensional structure of the MEV was first predicted using AlphaFold3 based on its amino acid sequence [52, 53], while experimentally determined crystal structures of the extracellular domains of TLR3 (PDB ID: 1ZIW), the TLR1/2 heterodimer (PDB ID: 2Z7X), and the TLR4–MD-2 complex (PDB ID: 4G8A) were retrieved from the Protein Data Bank and used as receptor inputs. All predicted models exhibited high quality, with pLDDT score above 70. The docking analysis and protein structures were prepared using the Schrödinger Protein Preparation Wizard. Bond orders were assigned, hydrogen atoms were added, zero-order bonds were assigned to metal ions when present, hydrogen bonding networks were optimized, and the structures were subjected to energy minimization using the OPLS_4 force field. Protein-protein docking was then performed using the Protein-Protein docking (PIPER) module implemented in Schrödinger under standard parameters. A total of 70,000 rotational ligand poses were sampled to ensure sufficient conformational exploration, and the top 30 docked conformations were retained. Among all generated poses, the top 1,000 solutions were clustered based on pairwise root mean square deviation (RMSD) of atomic positions. Docked complexes were ranked according to cluster population, and the representative structure from the most populated cluster was selected for subsequent analyses.

### Molecular dynamics simulation

Molecular dynamics (MD) simulations were performed to investigate the conformational stability and dynamic behavior of the protein-protein complexes using the Desmond module of the Schrödinger software package (Schrödinger 2023). Protonation states of amino acid residues were assigned using PROPKA 3.0, and all crystallographic water molecules were retained. The systems were constructed with the System Builder module and parameterized using the OPLS4 force field. Each complex was solvated in a cubic TIP3P water box with a 10.0 Å buffer and neutralized by adding Na^+^ or Cl^+^ ions. Energy minimization was performed using the steepest descent method for 50,000 steps, followed by NVT and NPT equilibration with positional restraints. The temperature and pressure were maintained at 300 K and 1 atm, respectively. Subsequently, 100 ns unrestrained MD simulations were carried out, with trajectory frames saved every 10 ps. Trajectory analyses, including root mean square deriation (RMSD), root mean square fluctuation (RMSF) and MM-GBSA binding free energy calculations, were performed using Schrödinger 2023.

### Immune response simulation

The C-ImmSim server (https://kraken.iac.rm.cnr.it/C-IMMSIM/index.php) is a computational model that mimics the dynamics of human humoral and cellular immune responses to infection, vaccines, and disease. Therefore, the innate and adaptive immune response to the MEV was simulated in humans using the C-ImmSim tool, with parameters including a random seed of 12345, a simulation volume of 10 µL, and a simulation time step of 100 (one time step=8 h). The MEV administered without lipopolysaccharide, was injected three times at intervals of 0, 14, and 28 days, respectively.

### In silico cloning and expression analysis of the MEV

To increase vaccine expression in mammals, codon optimization of the sequence was performed using the ExpOptimizer online tool (https://www.novopro.cn/tools/codon-optimization.html), with transcriptional and translational efficiencies assessed through GC content analysis and the codon adaptation index (CAI). Subsequently, the MEV was constructed by inserting the optimized fragments into a eukaryotic expression vector (pJW) with HindIII and BamHI restriction endonuclease sites at the 5’ and 3’ ends, respectively. The pJW eukaryotic expression vector (Kindly gifted by Dr. Yanmin Wan’s laboratory at Fudan University) was chosen for plasmid construction and protein expression based on its superior efficiency in protein production, as previously confirmed in our studies [54]. The MEV design was visualized using the SnapGene software. To analyze the expression of MEV protein, HEK-293T cells were transfected with p-MEV or pJW plasmid using lipofectamine 2000 for 48 h. After cells were collected and lysed, the cell lysates samples (30 g/lane) were separated by SDS-PAGE on 10% gels and transferred onto PVDF membranes. Subsequently, the membrane was blocked with 5% (w/v) nonfat milk in TBST buffer for 1 h at room temperature and incubated with primary antibodies mouse anti-His mAb (1:1000, Cat#TA-02, ZSGB-Bio) at 4℃ overnight. Afterward, the membrane was incubated with goat anti-mouse HRP-conjugated secondary antibody (1:5000, Cat#AS003, Abclonal) for 1 h at room temperature. Finally, the bands were visualized by enhanced chemiluminescent reagents (Cat#WBKLS0500, Merck).

## Results

### Identification of key upregulated DEGs in LUAD

**Fig. 2:**
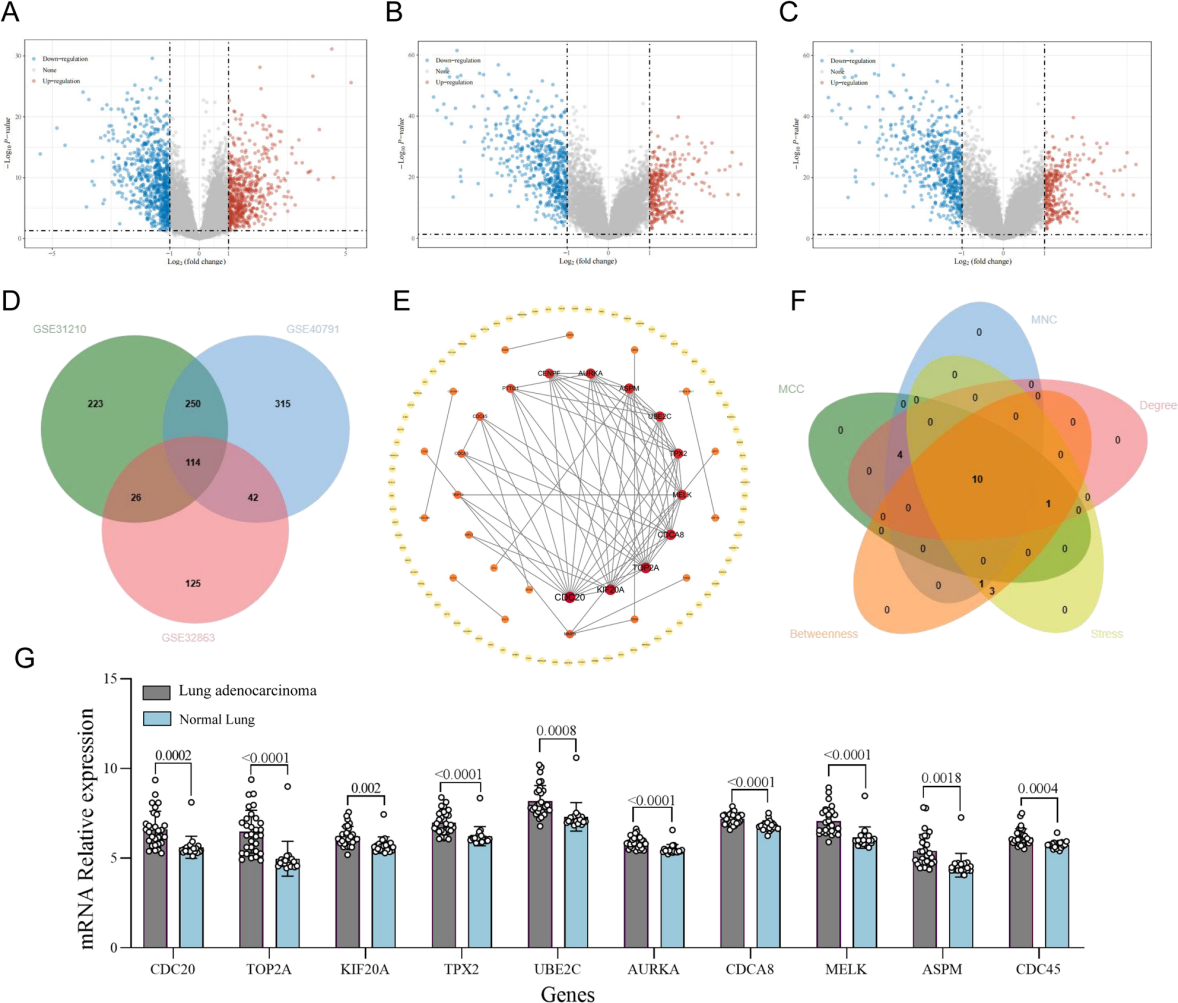
Screening of top 10 hub genes in lung adenocarcinoma. Volcano plot of DEGs based on the (**A**) GSE31210, (**B**) GSE40791, and (**C**) GSE32863 of GEO datasets, respectively; (**D**) The 613 upregulated DEGs in the GSE31210 dataset, 721 upregulated DEGs in the GSE40791 dataset, and 307 upregulated DEGs in the GSE32863 dataset were subjected to Venn diagram analysis. The coincident part represents the DEGs shared by the three datasets; (**E**) The PPI network of 114 upregulated DEGs in lung adenocarcinoma was analyzed by STRING tool and presented using Cytoscape software; (**F**) Venn diagram of overlap of 15 DEGs among 5 algorithms. **G** Validation of the differential expression of the top 10 hub genes in lung adenocarcinoma and normal lung tissue based on GSE31547. DEGs: differentially expressed genes

To identify the key genes in LUAD, gene expression profiles from three datasets were retrieved from the GEO database and DEGs were identified. As shown in the Volcano plot, in total, 613, 721, and 307 upregulated DEGs and 776, 1250, 591 downregulated DEGs were identified from the GSE31210, GSE40791, and GSE32863 datasets of LUAD, respectively (Fig. 2A - C). Subsequently, 114 up-regualted and 269 down-regulated genes were found to be common among the three datasets using a Venn diagram (Fig. 2D and Supplementary Figure S1). Because up-regulated genes are more likely to encode antigens accessible to vaccine induced immune responses, subsequent analyses focused on up-regulated genes, while down-regulated genes were excluded from further investigation.

### Hub gene identification and validation

The PPI network was analyzed by STRING to identify interacting proteins, encoded by 114 common DEGs and visualized by Cytoscape (Fig. 2E). Subsequently, interactions with the highest confidence scores (greater than 0.9) from the STRING database were utilized. To identify hub genes from the PPI network, the top 15 DEGs were identified using Cytoscape with five ranking algorithms including MCC, MNC, Degree, Stress, and Betweenness (Supplementary Figure S2). Subsequently, 10 shared hub genes were screened from 15 DEGs of each algorithm, including CDC20, TOP2A, KIF20A, TPX2, UBE2C, AURKA, CDCA8, MELK, ASPM, and CDC45 (Fig. 2F). To validate the results, the expression of the 10 hub genes was also evaluated in the GSE1547 dataset. We found that the expression of the top 10 hub genes was significantly increased in LUAD as compared to the normal lung tissue (Fig. 2G). Furthermore, a Kaplan-Meier plotter was used to assess the association between the expression levels of the identified hub genes and patient survival in LUAD. The results showed that increased expression of each of the 10 hub genes was negatively correlated with the overall survival rate of LUAD patients (Supplementary Figure S3). Finally, to better characterize the Hub gene roles, GO and KEGG pathway enrichment analyses were performed on these upregulated genes firstly. The GO biological process (BP) enrichment analysis revealed that the identified DEGs were predominantly enriched in mitotic spindle assembly checkpoint signaling, metaphase chromosome alignment, and sister chromatid separation, indicating dysregulation of key mitotic and cell cycle in LUAD. In contrast, no molecular function (MF) or cellular component (CC) terms reached statistical significance under the same adjusted P value threshold. KEGG pathway enrichment analysis further revealed significant enrichment in cancer relevant pathways including Cell cycle, Transcriptional misregulation in cancer and ECM–receptor interaction, supporting the involvement of these genes in tumor growth and invasion(Supplementary Figure S4A-4B).

Subsequently, GO and KEGG enrichment analyses were conducted specifically on the hub genes. GO biological process analysis revealed that hub genes were predominantly involved in cell cycle phase transition, mitotic spindle organization, metaphase/anaphase transition, and sister chromatid separation, further emphasizing their critical roles in mitotic regulation. Consistently, KEGG pathway analysis showed significant enrichment in cell cycle and ubiquitin-mediated proteolysis, indicating coordinated dysregulation of cell cycle control and cell cycle regulated protein degradation. Collectively, our data indicated that the 10 hub genes were highly expressed in LUAD and these enrichment patterns support that the identified hub genes function as central regulatory nodes driving LUAD proliferation and tumor progression (Supplementary Figure S4C-4D).

### Neoantigens, CTL and B cell epitopes prediction of hub genes

Under the criteria of neoantigens screening, 43 epitopes from 7 hub genes were screened using the TSNAdb online tool, including 41 SNV epitopes and two INDEL epitopes (Supplementary Table 1). Subsequently, CTL epitopes of hub genes were identified using the NetCTL1.2 server with 12 MHC I supertypes. Furthermore, the selected epitopes were refined for antigenicity, allergenicity and toxicity. 8 unique neoantigen epitopes with antigenicity, non-allergenicity, and non-toxicity were selected as CTL epitope of MEV construction (Table 1). Furthermore, the population coverage of the 8 selected epitopes was evaluated using the IEDB Population Coverage Analysis tool to assess their potential association with MHC-I molecules across global populations. The results showed that 8 selected epitopes exhibited measurable population coverage across the majority of countries analyzed. At the global level, the population coverage for MHC class I was 63.24%, while the highest coverage was observed in East Asia, reaching 72.01% (Supplementary Figure S5). These results indicate that the epitope set confers a moderate but meaningful breadth of population coverage. Additionally, immune checkpoint PD-L1 is a key regulator of immune responses, and antibodies targeting PD-L1 can restore T-cells function and immune activity against tumors by preventing interaction with PD-1. Therefore, the linear B-cell epitopes of the PD-L1 extracellular sequence (residues 19 to 238) were predicted using IEDB and the BCpreds online server. A total of nine linear B cell epitopes were identified by IEDB, whereas three epitopes were predicted by BCpreds tool. By comparing the results from both tools, two overlapping B-cell epitopes were identified, and thus two B cell epitopes (36-HQVLSGKTTTTNSKREEK-53 and 50-FRRLDPEENHTAEL-67) of human PD-L1 were selected for MEV construction. BLASTp analysis optimized for short peptide queries showed that, other than PD-L1, no additional human proteins exhibited both high sequence identity and full-length coverage with the selected PD-L1 B-cell epitopes. The detected matches showed partial coverage or corresponded to intracellular proteins, indicating a low risk of non-specific antibody cross-reactivity.

**Table 1:** 8 CTL epitopes were selected with antigenicity, non-allergenicity and non-toxicity

Gene	Name	Peptide	MHC binding affinity	Rescale binding affinity	C-terminal cleavage affinity	Transport efficiency	Toxin Prediction	Allertop Prediction	Antigenicity
CDC20	CTL-1	RLRNMTSHF	0.5495	1.0908	0.9576	2.845	No	No	Yes
CDC20	CTL-2	SHFARVGSL	0.3519	1.1267	0.9639	1.125	No	No	Yes
ASPM	CTL-3	VRQIKRIHF	0.3796	1.0091	0.9337	2.769	No	No	Yes
ASPM	CTL-4	NSMHSSATL	0.3201	1.0249	0.9588	1.095	No	No	Yes
TPX2	CTL-5	TYVHLAQQV	0.4426	0.9425	0.7837	0.634	No	No	Yes
ASPM	CTL-6	IQSAFRIAK	0.4649	0.8751	0.6109	0.557	No	No	Yes
KIF20A	CTL-7	LALQRSQRL	0.1561	0.5332	0.9445	1.027	No	No	Yes
ASPM	CTL-8	FRRDNMEEI	0.1323	0.4518	0.8234	0.693	No	No	Yes

### MEV construction and Secondary and tertiary structural prediction

**Fig. 3:**
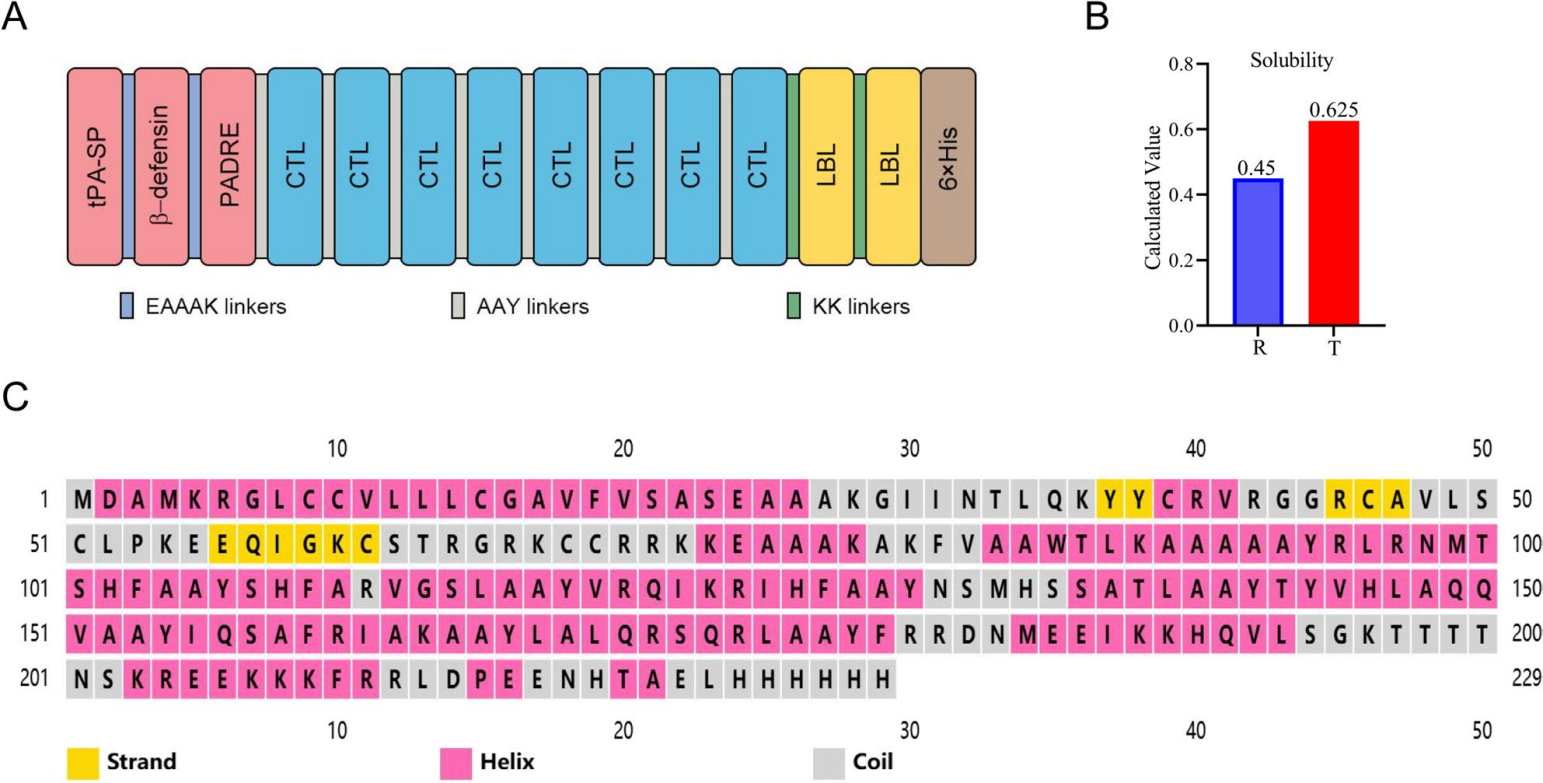
Design, solubility, and secondary structure of the multi-epitopes vaccines. **A** Schematic diagram of multiple epitopes vaccine construction. **B** Solubility of the mult-epitope vaccine predicted by the Protein-Sol server. The letter of R and T represent the reference protein and designed vaccine protein, respectively. **C** The secondary structure of the multi-epitope vaccine was predicted by the PSIPRED server

**Fig. 4:**
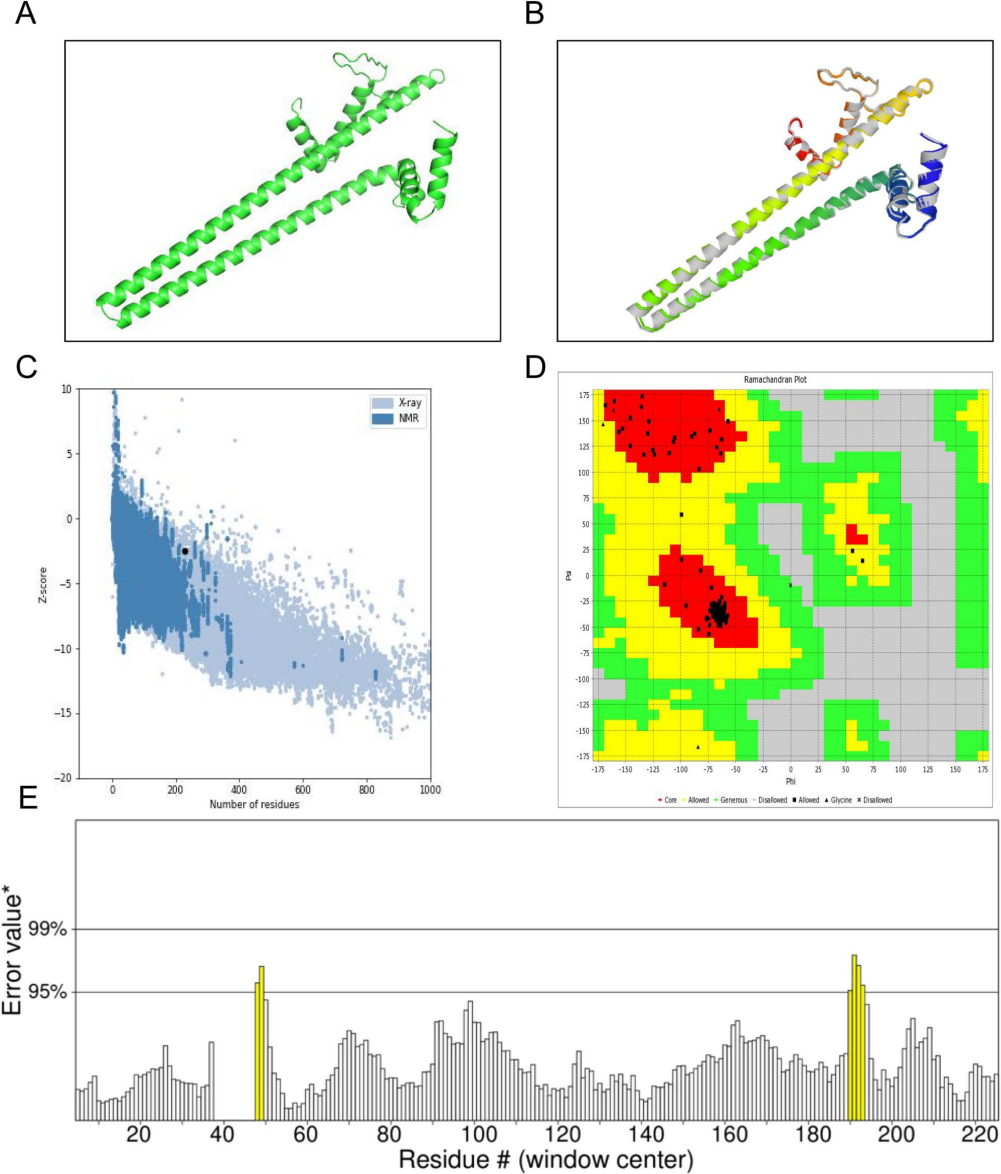
Tertiary structure validation and refinement of MEV. **A** the Tertiary structure of the MEV was predicted by the 3Dpro server. **B** Tertiary structure of the MEV was refined by the GalaxyRefine web server. **C** ProSA-web analysis of the refined MEV. Z-scores of all protein chains was shown in the PDB determined by X-ray crystallography (light blue dots) and NMR spectroscopy (dark blue dot). The Z-score of the refined vaccine is highlighted with a black dot. **D** ERRAT analysis of the refined MEV. Regions with low error values are represented by white bars. Regions with an error value between 95% and 99% are represented by yellow bars. **E** Ramachandran analysis showed the percentage of amino acids in favored region, outlier regions, and rotamer regions of the refined vaccine. The most favored areas of amino acids are represented by darker color

To design the MEV against LUAD, eight CTL epitopes and two B-cell epitopes were joined by AAY and KK linkers, respectively (Fig. 4A). Additionally, PADRE was chosen and incorporated into the N-terminus of the CTL epitopes with an AAY linker. Moreover, to increase the immunogenicity of the MEV, β-defensin was integrated into the MEV with an EAAAK linker. The antigenicity analysis revealed that the MEV exhibited high antigenicity, with scores of 0.62 and 0.58 using the VaxiJenv2.0 and ANTIGENpro servers, respectively. The allergenicity analysis indicated that the MEV was non-allergenic based on both the AllerTOPv.2 and AllergenFP servers. Simultaneously, the physiochemical characteristics of the MEV were also evaluated using Expasy ProtParam (Table 2). The final MEV construct consisted of 229 amino acids, and its theoretical molecular weight was 25.8 kDa. The isoelectric point of the MEV was 10.11, indicating a positively charged under physiological conditions. The instability and aliphatic index of MEV were found to be 39.32 and 75.24, respectively, indicating that the MEV is a stable and highly thermostable protein. The estimated half-life of MEV was 30 h in vitro, 20 h in yeast and 10 h in E. coli, suggesting its stability in vitro and in vivo. The Grand Average of Hydropathicity (GRAVY) score of −0.355 suggested that the vaccine is hydrophilic. The predicted solubility score of the MEV by Protein-sol was 0.625, suggesting its solubility when expressed in host expression system (Fig. 3B). The secondary structure of the MEV was predicted using PSIPRED v3.3 and the RaptorX Property online server. As shown in Fig. 3C, the secondary structure of the MEV contains 63% alpha-helices, 32% random coils, and 3% extended strands.

**Table 2:** Characteristics of the MEV construct

Characteristics	Assessment	Servers
Antigenicity	0.6215	VaxiJen
	0.585192	ANTIGENpro
Allergenicity	NON-ALLERGEN	AllerTOP v2.0
	NON-ALLERGEN	AllergenFP v.1.0
Number of amino acids	229	Expasy Protparam
Molecular Weight	25834.01	
Theoretical PI	10.11	
Estimated half-life(Hour)	30 h (mammalian reticulocytes, in vitro). >20 h (yeast, in vivo). >10 h (Escherichia coli, in vivo).	
Instability index	39.32	
Aliphatic index	75.24	
GRAVY	−0.355	

### Tertiary structural refinement and validation

The 3D structure of the MEV model was initially predicted by the 3Dpro server (Fig. 4A). The initial MEV model was refined using the GalaxyRefine web server. Five optimized models of the MEV were produced by GalaxyRefine and ranked by GDT-HA and MolProbity values (Table 3). High GDT-HA values, low RMSD values, and low MolProbity scores indicate a high-quality protein structure model. Based on these parameters, the optimized model 4, with GDT-HA (0.8941), RMSD (0.539) and MolProbity (1.544) was selected for further analysis (Fig. 4B). The quality and potential errors of the initial and refined MEV 3D model were evaluated by ProSA-web, ERRAT, and Ramachandran plots. After refinement by ProSA, the Z-score of the MEV model improved from − 2.01 to −2.47, which falls within the acceptable range for native proteins (Fig. 4C). Based on ERRAT analysis, the overall-quality factor of the MEV model was increased from 80.9302 to 97.1564, reducing potential errors and unreasonable regions (Fig. 4D). The refined model was further evaluated for residue stereochemical qualities using Ramachandran plot analysis. The results showed that approximately 79% of the residues were located within the favorable regions, 20.4% in additionally allowed regions, 0.5% in generously allowed regions, and only 0.1% in disallowed regions (Fig. 4E). These data demonstrated that the overall quality of the 3D model was improved after refinement by the GalaxyRefine server.

**Table 3:** 5 models refined by the Galaxy web server

Model	GDT-HA	RMSD	MolProbity	Clash score	Poor rotamers	Rama favored
Initial	1	0	3.049	84.2	4.2	96.9
MODEL 1	0.881	0.57	1.564	11.2	0.6	100
MODEL 2	0.893	0.554	1.544	10.6	0.6	99.6
MODEL 3	0.8712	0.597	1.524	10.1	0.6	99.6
MODEL 4	0.8941	0.539	1.544	10.6	0.6	99.6
MODEL 5	0.869	0.599	1.596	11.2	1.1	99.6

*GDT-HA* Global distance test-high accuracy, *RMSD* Root mean square deviation, *Rama favored* Ramachandran favored score, *Clash score* the number of atomic clashes per 1000 atoms, *Poor rotamers* the percentage of rotamer outliers

### Prediction of discontinuous B cell epitopes

**Fig. 5:**
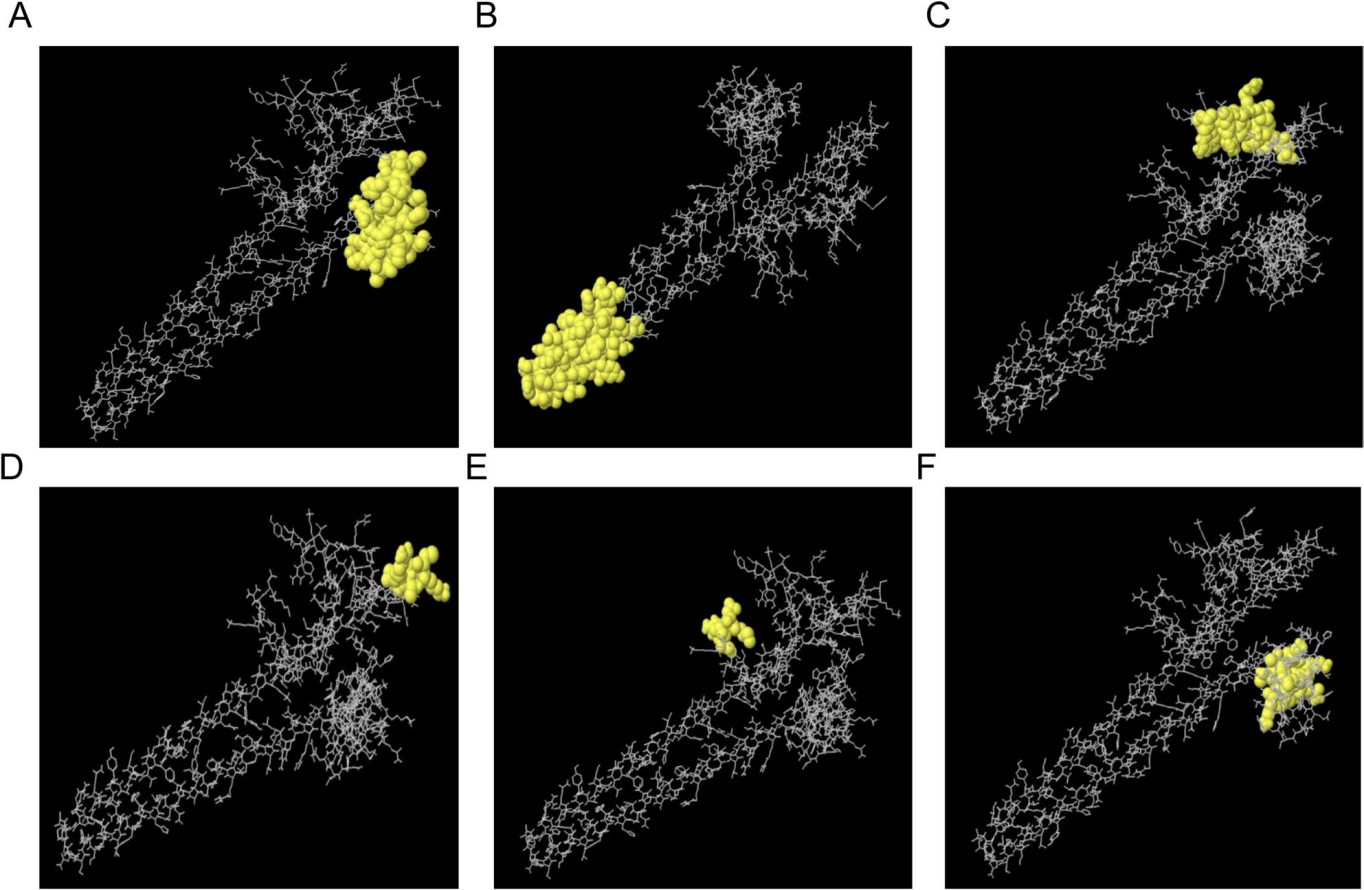
Discontinuous or conformational B-cell epitopes predicted by the ElliPro server. **A**-**F** Yellow balls represent the conformational B-cell epitopes, and the bulk of the polyprotein is represented by gray sticks

A discontinuous B-cell epitope is the antigenic region within a protein that can interact directly with the B-cell receptor to trigger humoral immunity [55]. The discontinuous B cell epitopes of the MEV were predicted using the ElliPro server. Six high quality discontinuous B cell epitopes were identified, comprising 24, 38, 18, 8, 4 and 12 amino acid residues, with corresponding scores of 0.866, 0.767, 0.659, 0.642, 0.58 and 0.573, respectively (Supplementary Table 2). The spatial position of six discontinuous B cell epitopes was presented as yellow balls in a 3D model of the MEV (Fig. 5). Notably, several residues within the selected linear PD-L1 B-cell epitopes (aa 36–53 and aa 50–67) overlapped with, or were spatially adjacent to, residues identified in the predicted discontinuous B-cell epitopes (including Y37, R40, aa 42–50, P53, and aa 57–64). This observation supported that the selected linear epitopes contribute to surface exposed antigenic regions in the 3D structure.

### Molecular docking

**Fig. 6:**
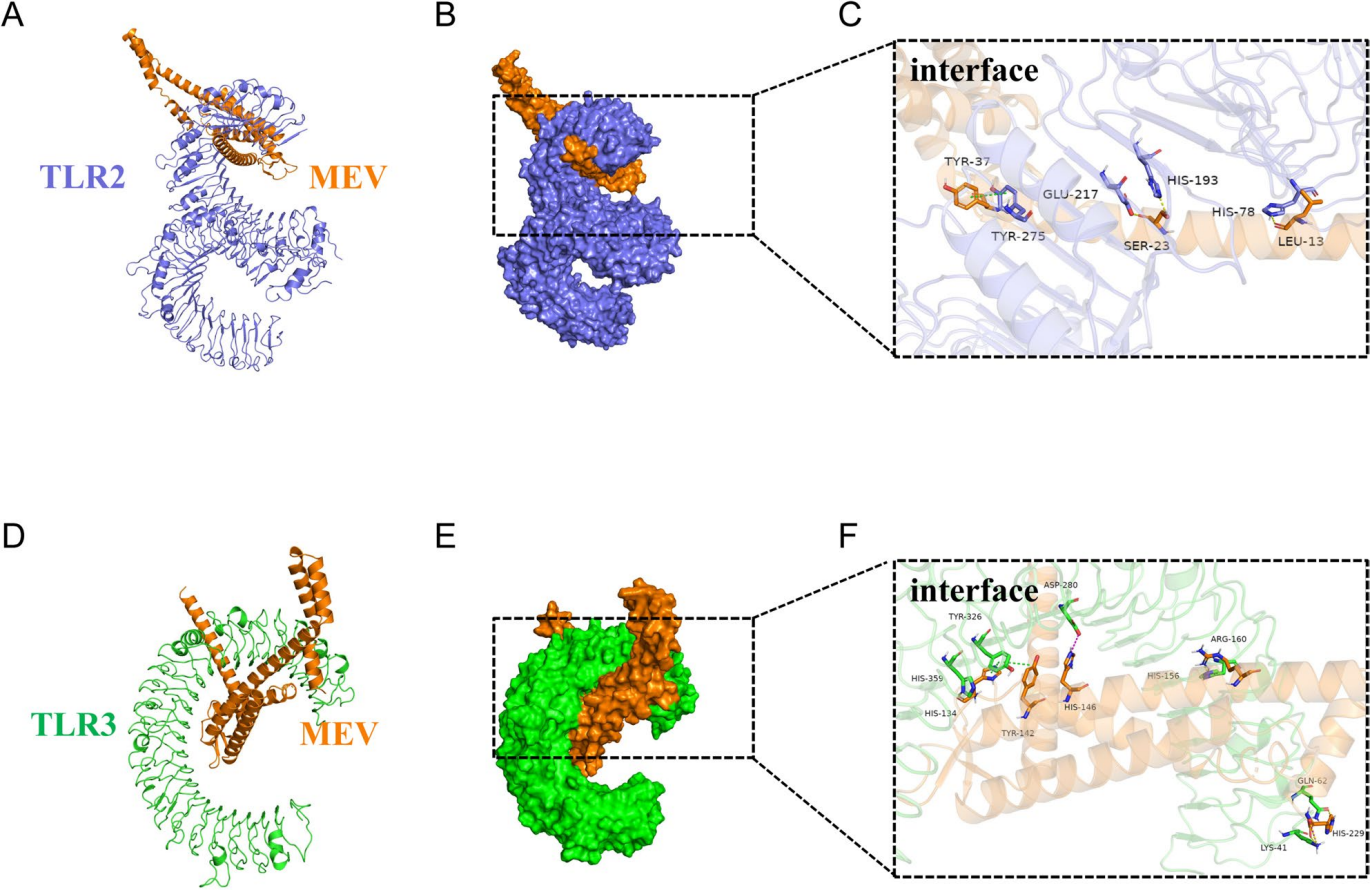
Molecular docking analysis of the MEV-TLR2 and MEV-TLR3 complex. **A** A cartoon depiction of the docked MEV-TLR2 complex, with TLR2 displayed in purple and the MEV construct in orange; (**B**) The surface depiction of the MEV-TLR2 complex; (**C**) The interaction interface between MEV and TLR2. **D** A cartoon depiction of the docked MEV-TLR3 complex, with TLR3 displayed in green and the MEV construct in orange; (**E**) The surface depiction of the MEV-TLR3 complex; (**F**) The interaction interface between MEV and TLR3. Interacting amino acid residues are shown as stick models. At the interaction interface, hydrogen bonds, salt bridges, and π-π stacking interactions are indicated by yellow, magenta, and green dashed lines, respectively

The three-dimensional structures of the MEV vaccine construct and the TLR2 and TLR3 receptors were first predicted using AlphaFold3, and protein-protein docking was subsequently performed using the Protein-Protein Docking (PIPER) module implemented in the Schrödinger software. The docking-based interaction modeling generated 30 clustered conformations for each MEV-TLR complex, which were ranked according to maximum cluster population. Interactions between the MEV vaccine construct and TLR2, TLR3, and TLR4 were subsequently evaluated. For the highest-ranked cluster, the maximum cluster sizes were 114 for TLR2, 88 for TLR3, and 96 for TLR4, with corresponding minimum energy scores of − 56.3, − 68.62, and − 44.58 kcal/mol, respectively. Structure inspection revealed distinct interaction pattern between MEV and the different Toll-like receptors. In the TLR2-MEV complex, TLR2 forms 3 hydrogen bonds with MEV and one *π-π* interaction(Fig. 6A - C). For TLR3-MEV complex, TLR3 established four hydrogen bonds and two salt bridge and two *π-π* interaction (Fig. 6D - F). In the case of TLR4-MEV complex, TLR4 forms two hydrogen bonds without detectable salt bridge formation(Supplementary Figure S6 and Supplementary Table 3). Collectively, these results demonstrate receptor specific interaction between MEV and TLR2, TLR3, and TLR4, highlighting distinct structural modes of potential innate immune receptor engagement.

### Molecular dynamic simulation

**Fig. 7:**
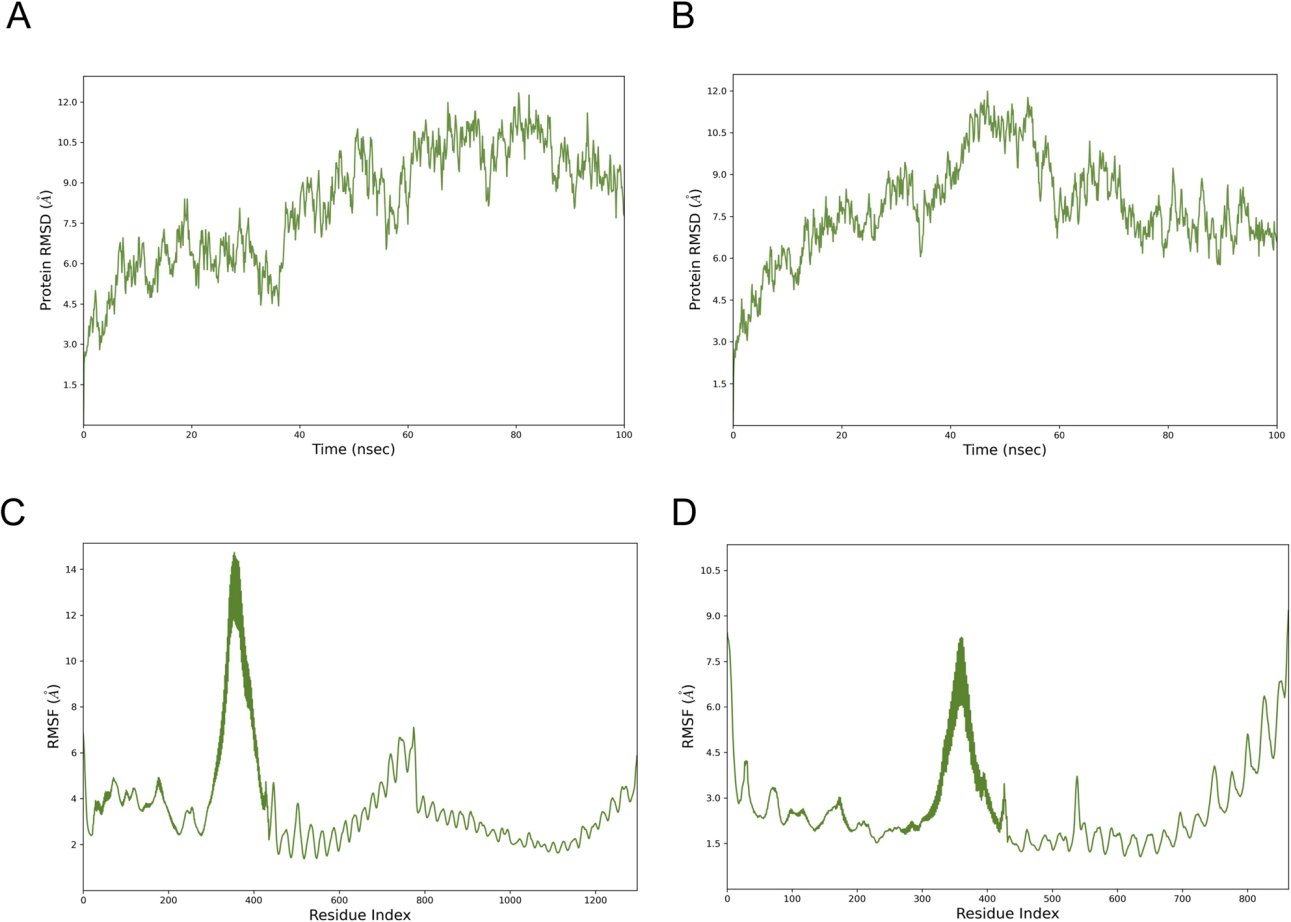
RMSD and RMSF plots to evaluate the residual stability and fluctuations of MEV-TLR2 and MEV-TLR3. **A** RMSD plot of MEV-TLR2; (**B**) RMSD plot of MEV-TLR3; (**C**) RMSF plot of MEV-TLR2; (**D**) RMSF plot of MEV-TLR3

The structural stability of the MEV-TLR2 and MEV-TLR3 complexes was first evaluated by calculating the RMSD over a 100 ns molecular dynamics simulation (Fig. 7A and B). For the MEV-TLR2 complex, the RMSD increased rapidly during the initial equilibration phase and continued to rise gradually, followed by sustained fluctuations at later stages of the simulation, indicating conformational adaptation and dynamic stability of the complex. In contrast, the MEV-TLR3 complex exhibited a noticeable RMSD fluctuations during the early stage of the simulation and reaching a maximum around the mid-simulation. After approximately 60 ns, the RMSD values decreased and stabilized with narrower fluctuations, indicating that the complex underwent structural relaxation and converged toward a stable binding conformation.

Residue level flexibility was further assessed by analyzing the RMSF profiles derived from the simulation trajectories of the protein-ligand complexes (Fig. 7C and D). For the MEV-TLR2 complex, most residues displayed relatively small fluctuations, indicating overall structural stability, while higher flexibility was mainly confined to loop regions, particularly between residues 300–400. Similarly, the MEV-TLR3 complex demonstrated overall reduced residue fluctuations with localized peaks in flexible regions, whereas interface associated residues remained comparatively stable. These results suggest that complex formation limits excessive residue mobility at the interaction interface in both systems. Binding free energy calculations were performed to further evaluate the energetic stability of the MEV-TLR complexes (Supplementary Table 4). Both the MEV-TLR2 and MEV-TLR3 complexes exhibited favorable total binding free energies (ΔG_bind), indicating energetically stable interactions. Energy decomposition analysis revealed that van der Waals interactions and lipophilic contributions were the dominant favorable forces driving complex formation, reflecting good structural complementarity at the binding interface. Hydrogen bonding provided additional stabilizing contributions, whereas electrostatic interactions were partially offset by polar solvation effects, consistent with the characteristic electrostatic-solvation compensation observed in MM-GBSA analyses. Overall, these results support the stable binding of MEV to both TLR2 and TLR3 from an energetic perspective.

### Immune response simulation analysis of the MEV vaccine

**Fig. 8:**
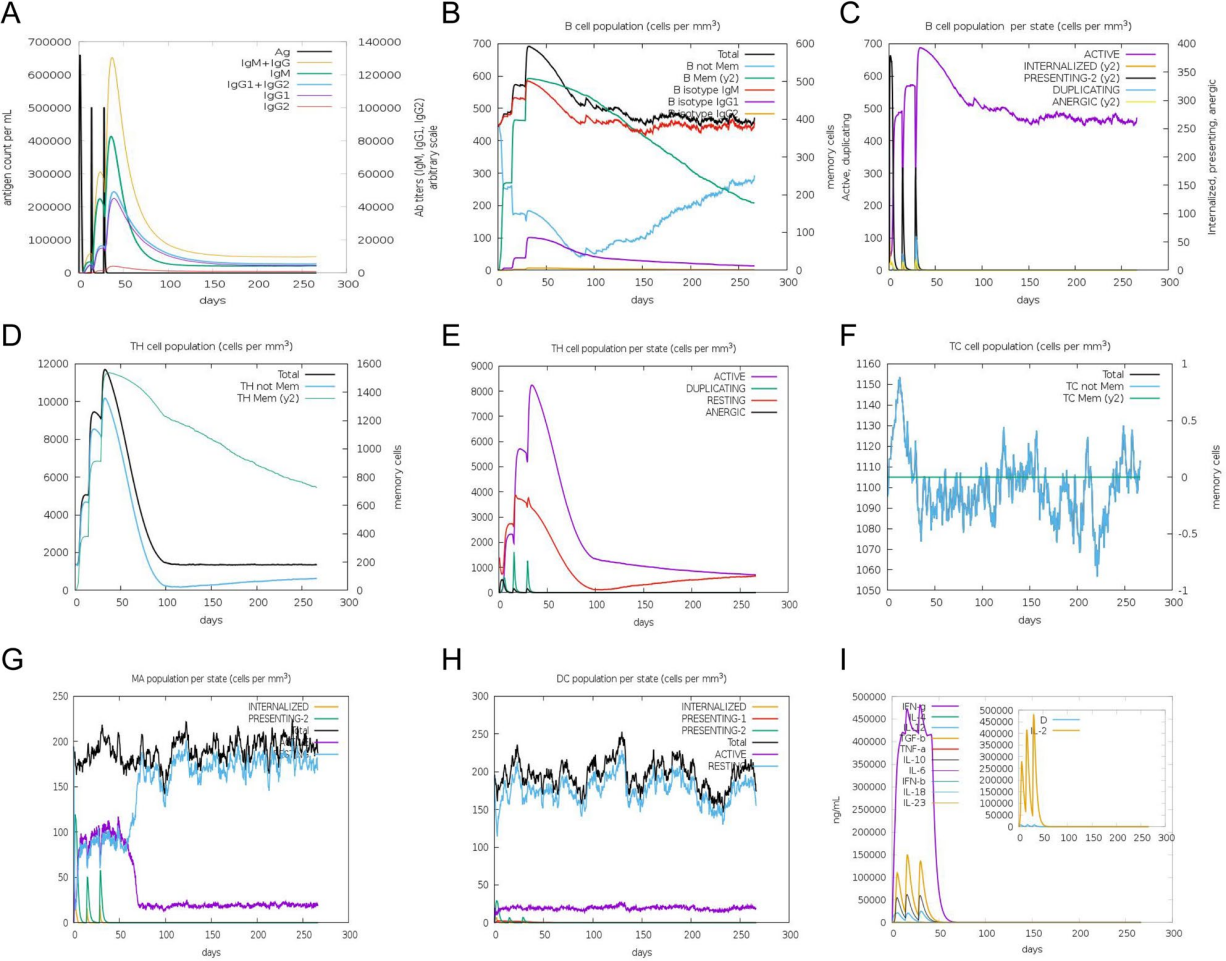
Immune simulation analysis of the MEV by the C-ImmSim server. **A** Antibody levels following vaccine injection (antigen is shown as a black line). **B** Memory B-cell, non-memory B-cell, and B-cell isotype populations. **C** Active B-cell population. **D** Memory T-helper and non-memory Th cell populations. **E** Active Th cell populations. **F** Memory cytotoxic T and non-memory Tc cell populations. **G** Active macrophage populations. **H** Active dendritic cell populations. **I** Cytokines production

The innate and adaptive immune simulation analysis was performed by simulating the MEV injection process through the C-ImmSim server. In silico analysis, it was found that MEV vaccination induced a significantly stronger MEV-specific antibody response. After the third immunization, the antibody titers of IgM, IgG1 and IgM + IgG reached a peak and then decreased gradually (Fig. 8A). Accordingly, the population of total B cells, memory B cells, and IgM + B cells reached a peak rapidly and persisted for a long time(Fig. 8B). Additionally, the population per state of active B cells, Th memory cells and active Th cells reached a peak and persisted for a long time (Fig. 8C - E). Antigen presenting cells and activated CTLs are essential for the host immune system to detect and kill tumor cells. As shown in Fig. 7F and G, the total populations of macrophages and dendritic cells (DC) were increased after the third immunization, indicating that antigen-presenting cells are sufficiently able to process and deliver antigens to T cells. In response to the antigen presentation, the population of presenting-2 of macrophages and DCs reached a peak after the first immunization (Fig. 8G and H). Moreover, the population of T-cytotoxic but not memory T cells showed strong responses after immunization (Fig. 8F). Finally, we found that the levels of cytokines such as IFN-γ, IL-2, TGF-β, IL-12, and IL-10 increased rapidly after each dose, indicating that the MEV had induced a favorable immune response (Fig. 8I).

### In silico cloning and expression verification of the MEV

**Fig. 9:**
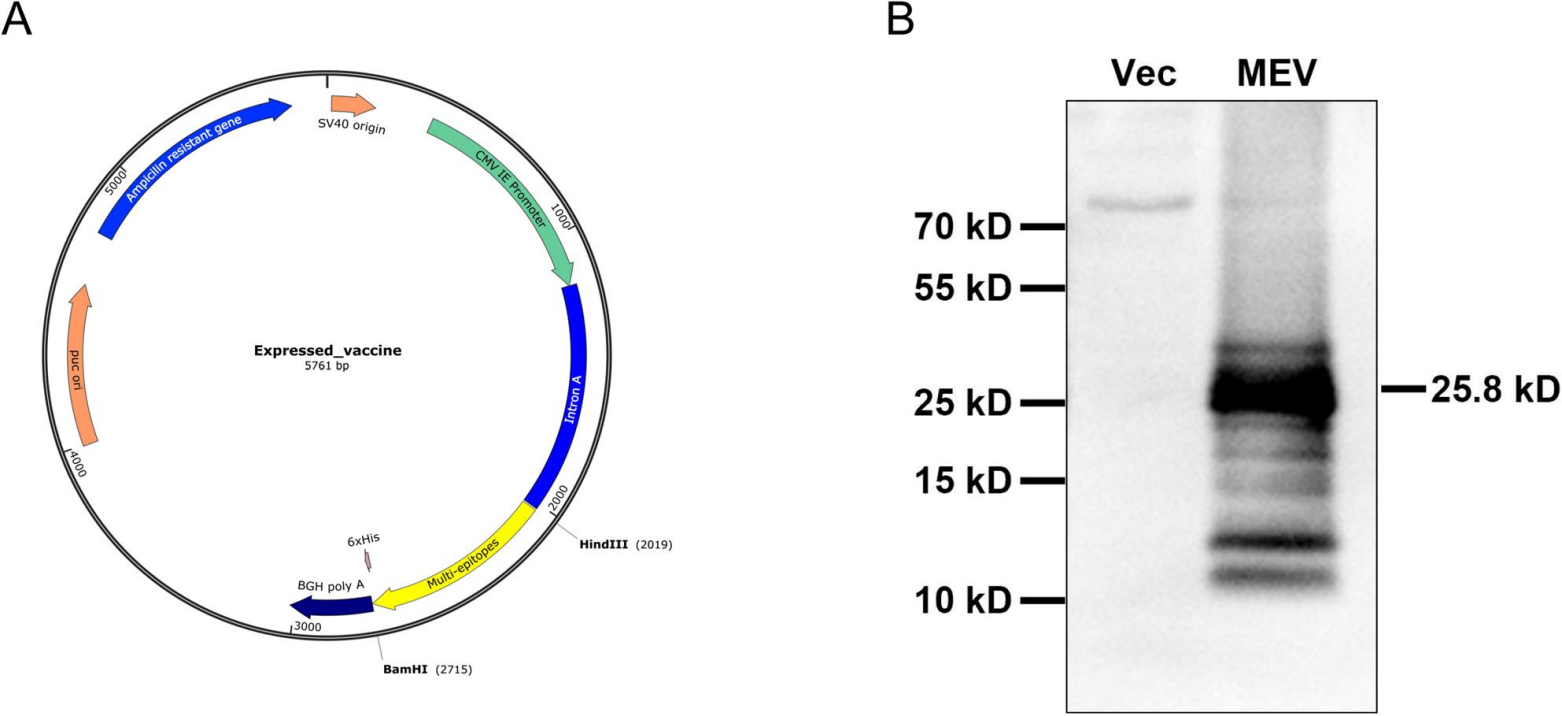
In silico cloning and expression verification of the MEV. **A** A multi-epitope fragment with human codon optimization was inserted into a eukaryotic expression vector. The yellow fragment represents the multi-epitope sequence. **B** The expression of the recombinant MEV plasmid was analyzed in HEK-293T cells by western blotting with a mouse anti-poly-histidine mAb

For the efficient expression of the MEV in mammals, the nucleotide sequence of the MEV was optimized according to the preference for human codon usage and sub-cloned into a eukaryotic expression vector (pJW) and visualized with SnapGene software. The GC content and CAI for the optimized MEV sequence were 54.15% and 0.82, respectively. Typically, a CAI above 0.8 and a GC content range from 30% to 70% are considered optimal for efficient protein expression. Finally, 687 nucleotides of the MEV fragment were inserted into the pJW vector using the HindIII and BamHI restriction endonucleases at the N-terminal and C-terminal, respectively (Fig. 9A). Meanwhile, the expression of the recombinant MEV plasmid was analyzed in HEK-293T cells by western blotting with a mouse anti-poly-histidine mAb. The results showed that a specific band at around 25 kDa on the PVDF membrane, indicating that the recombinant MEV protein was expressed in HEK-293T cells (Fig. 9B).

## Discussion

MEVs based on neoantigens have distinct advantages over traditional vaccines, such as, overcoming immune tolerance, covering a wide range of target antigens, and reducing side effects [12, 56]. Recently, vaccines incorporating shared neoantigens derived from high frequency mutations of oncogenes across multiple cancers have emerged as a promising immunotherapy for LUAD [26, 28, 57]. However, inadequate immunogenicity and the immunosuppressive tumor microenvironment remain major challenges that impede their clinical efficacy. To enhance the vaccine immunogenicity, we refocused on the source of tumor neoantigen epitopes. Unlike the conventional tumor neoantigen discovery strategy that prioritizes epitopes derived from recurrently mutated genes, we systematically interrogated tumor neoantigen epitopes from hub genes. Network central hub genes, operating as critical regulators within biological networks, orchestrate cross-talk between oncogenic signaling cascades [58]. A series of studies have shown that hub genes are significantly associated with overall survival and serve as therapeutic targets in lung cancer [59–61] and have also been proposed as promising candidates for mRNA vaccine development [61, 62]. Building on this rationale, our MEV is designed as an active immunization strategy intended to elicit antigen specific immune responses against hub genes, and thus may be conceptually complementary to checkpoint inhibition. Our strategy leverages hub genes that are consistently up-regulated and topologically central across multiple independent LUAD cohorts, this strategy improves the robustness and generalizability of antigen and may potentially provide broader immune coverage. The final MEV construct integrates hub gene derived CTL epitopes with immunomodulatory components, including PD-L1 derived B cell epitopes, PADRE, and β-defensin. This design distinguishes our MEV from prior LUAD vaccine approaches that typically target single shared antigens, such as MUC1, MAGE-A3, or PRAME based vaccines [63–65], as well as from fully personalized neoantigen peptide vaccines that require patient specific sequencing and manufacturing [66]. Importantly, by utilizing the lower immune tolerance of neoantigens, the proposed MEV strategy helps overcome central and peripheral immune tolerance that often limits the efficacy of tumor associated antigens. Therefore, we propose hub gene driven MEV framework as a complementary immunotherapeutic strategy for LUAD, with the potential to enhance CTL infiltration, activation and immune modulation at both cellular and humoral immunity.

To identify robust hub genes, upregulated lung cancer associated genes were screened from GEO datasets and subsequently analyzed using PPI networks combined with a consensus-based intersection strategy across multiple cytoHubba algorithms. Similar approaches have been applied in PPI related network studies using cytoHubba to reduce algorithm specific bias and screen high confidence candidates [67–69]. Although this strategy is conservative and may exclude some potentially relevant genes, the selected hub genes were further validated by an independent dataset and survival analyses, providing additional evidence for their relevance. Using integrative bioinformatics analysis of three independent GEO datasets, we identified 10 topologically hub genes (CDC20, TOP2A, KIF20A, TPX2, UBE2C, AURKA, CDCA8, MELK, ASPM and CDC45) in LUAD. Kaplan-Meier survival curve analysis demonstrated significantly reduced overall survival in patients with high expression of these hub genes suggesting that they could be promising therapeutic candidates for LUAD. In addition, KEGG enrichment analysis of the hub genes revealed significant enrichment in cell cycle, ubiquitin-mediated proteolysis, and oocyte meiosis pathways, highlighting their involvement in cell cycle regulated protein degradation and mitotic control, consistent with their central roles in LUAD progression. Notably, these findings are well supported by the functional enrichment patterns observed in the upregulated DEGs, which were predominantly enriched in mitotic spindle checkpoint signaling, chromosome alignment, and sister chromatid separation, collectively indicating widespread dysregulation of cell cycle and mitotic processes in LUAD. Beyond their established roles in tumor proliferation, these hub genes have also been linked to immune escape and tumor microenvironment remodeling, including altered immune cell infiltration and T cell activity [70–72]. From this perspective, screening neoantigens derived from these hub genes may provide a rational strategy to improve antigen immunogenicity, overcome immune tolerance, and enhance the translational potential of multi-epitope vaccines for LUAD. Although RNA sequencing based LUAD transcriptomic datasets provide higher resolution, this study focused on microarray-based cohorts to allow for multiple dataset integration and cross validation among independent populations. Future studies integrating RNA sequencing data will be important to further refine and validate these findings.

TSNAdb v2.0 is a systematically immuno-oncology platform designed to provide high-confidence tumor-specific immunogenic epitopes for neoantigen-based therapeutic development [73]. We systematically identified tumor-specific neoantigen candidates derived from LUAD hub genes using TSNAdb v2.0. Subsequently, we predicted CTL epitopes targeting 12 HLA-I subtypes using NetCTL1.2. Following rigorous immunoinformatic screening, eight CTL epitopes with non-toxic, non-allergenic, and antigenic properties were identified from CDC20, ASPM, TPX2 and KIF20A.These hub genes have been widely reported to play crucial roles in lung cancer proliferation and metastasis [74–76]. Following epitope screening, future studies may incorporate network level computational strategies, such as integrating protein–protein and protein–metabolite interaction analyses, to expand the identification of potential vaccine targets [77]. In parallel, hybrid approaches for predicting the IFN-γ induction potential of CTL epitopes could be applied as complementary tools to further refine multiepitope vaccine design [78]. Previous studies revealed that the population coverage score of more than 60% was considered as an acceptable population coverage [79–81]. Similarly, the selected MHC class I–restricted epitopes exhibited an estimated population coverage of 63.24% worldwide, suggesting a moderate breadth of coverage. It should be acknowledged that population coverage serves as a supportive rather than definitive metric of vaccine breadth, and that additional evidence will be required to further validate this conclusion.

The immunosuppressive tumor microenvironment is another obstacle for therapeutic cancer vaccines as it creates barriers to effective immune activation and effector cell infiltration [82]. However, emerging evidence demonstrates that combination therapies integrating cancer vaccines with ICIs can overcome these limitations, leading to favorable clinical outcomes [83–86]. Cancer vaccines can elicit tumor-specific T cells in the periphery or in situ tumor and facilitate their trafficking into the tumor microenvironment, thereby enhancing tumor-infiltrating lymphocyte (TIL) infiltration [87]. Concurrently, ICIs such as anti-PD-1/PD-L1 and anti-CTLA-4 antibodies alleviate T cell exhaustion by disrupting inhibitory receptor-ligand interactions, thereby amplifying the effector functions of TILs [88]. In addition, it is well recognized that immune associated modules are frequently identified in gene co-expression network analyses. Large scale cross tissue studies have demonstrated that immune related modules are present in multiple human tissues, including liver, lung, kidney cortex, uterus, and adipose tissue, supporting the biological relevance of co-expression defined immune networks [89]. Based on this evidence, linking co-expression derived hub genes to immune regulatory pathways, such as immune checkpoint signaling, represents a biologically plausible strategy. Therefore, two PD-L1 B-cell epitopes were incorporated into the MEV. Furthermore, critical immunogenic components were added to the MEV to enhance efficacy. Suitable linkers were strategically introduced to connect epitopes and functional domains, ensuring structural integrity while minimizing junctional immunogenicity. Recent immunoinformatics based vaccine studies emphasize that effective multi epitope vaccine design relies not only on antigen selection, but also on the rational integration of immune stimulatory components to enhance overall immunogenicity [90]. In line with this concept, our vaccine construct was designed within an immunologically optimized framework rather than through simple epitope concatenation. β-defensins function as endogenous adjuvants that bridge innate and adaptive immunity within the tumor immune microenvironment [91]. Mechanistically, β-defensins enhance the expression of inflammatory cytokines and chemokines in macrophages and promote the maturation and activation of antigen presenting cells, thereby increasing antigen immunogenicity. In addition, β-defensins can directly exert cytotoxic effects on tumor cells, independent of immune receptor signaling, leading to the release of damage associated molecular patterns (DAMPs) and tumor associated antigens (TAAs) [92–94]. Importantly, as naturally occurring human peptides, β-defensin has emerged as a promising molecular adjuvant candidate that can enhance antigen-specific immunity in multiple animal models and in silico vaccine designs without obvious safety concerns to date, suggesting potential safety advantages [95, 96]. Physicochemical characterization revealed favorable biophysical properties critical for vaccine stability and manufacturability, including a molecular weight of 25.8 kDa (theoretical pI: 10.11), high solubility (Protein-Sol score: 0.625), thermal stability (aliphatic index: 75.24; instability index: 39.32), and an extended half-life (> 10 h). Immunogenicity profiling confirmed strong antigenicity (VaxiJen score: 0.62) with non-allergenicity and non-toxicity. Structural validation via GalaxyRefine optimization yielded a refined 3D model with high accuracy (GDT-HA: 0.8941; RMSD: 0.539 Å; MolProbity score: 1.544), further supported by ERRAT (97.1564%), Ramachandran favored regions (79%), and a ProSA-web Z-score (−2.47) within native protein ranges. The expression efficiency of the codon-optimized MEV construct was further validated in mammalian host systems.

The vaccine elicited protective immunity by robustly activating both humoral and cellular immune responses. B lymphocyte mediated humoral immunity was predominantly driven by discontinuous B cell epitopes within the vaccine [97]. Consistently, six discontinuous B cell epitopes within the MEV were computationally identified using the ElliPro server (IEDB), validating the MEV’s capacity to induce potent antibody mediated immune responses. Previous studies have shown that the ability of TLR agonists to activate DC maturation and cytokine production facilitates the connection between the innate and adaptive immunity, leading to the activation of CTLs that eliminate tumor cells [51, 98]. Similarly, the predicted interaction models offer valuable structural insights into how the multi-epitope vaccine construct interacts with multiple Toll-like receptors (TLRs). The formation of energetically favorable docking complexes supports the ability of MEV to bind multiple innate immune receptors. The receptor specific interaction patterns observed among TLR2, TLR3, and TLR4 are reflected by distinct interaction interfaces and bonding features. These differences support a model in which MEV may interface with multiple innate immune signaling, thereby potentially facilitating coordinated activation of downstream adaptive immune responses. The stability of this complex was further validated through molecular dynamics simulations using RMSD and RMSF analyses, supporting a dynamically stable interaction between the vaccine construct and the receptor. To evaluate the immunogenicity of the MEV, the C-ImmSim server, an agent-based immune simulator, was used to model the immune response elicited by a three-dose vaccination regimen administered at two-week intervals. The computational analysis revealed robust humoral and cellular immune activation, characterized by elevated IgM/IgG titers, expansion of memory B cells, and increased populations of Th cells, CTLs, macrophages, and DCs as well as increased cytokines production. Notably, the simulated three-dose immunization schedule mirrors commonly used clinical vaccination regimens, further supporting the translational relevance of the predicted immunogenicity profile. Beyond experimental validation, the hub gene driven MEV design proposed in this study may have broader translational implications. By selecting neoantigens derived from functionally central and dysregulated genes shared among multiple patient cohorts, this strategy may serve as a shared or semi-personalized vaccine platform that complements immune checkpoint blockade. Moreover, with the increasing availability of patient level transcriptomic and genomic data, hub gene prioritization may be integrated into personalized vaccine design pipelines. Given that hub genes frequently represent conserved regulatory nodes across multiple cancer, this framework may also be applied to other solid tumor types, which should be further explored in future studies.

Several limitations of this study should be acknowledged. First, the identification and selection of T cell epitopes and B cell epitopes were based on integrative immunoinformatics analyses. Although multiple computational criteria, including tumor specificity, antigen processing and presentation and population coverage, were used to support their potential immunogenic relevance, these predictions remain indirect and cannot establish definitive epitope immunogenicity without experimental validation. Second, the immune response profiles predicted by the C-ImmSim server are derived from computational models and may not fully simulate the complexity, heterogeneity, and regulatory dynamics of in vivo immune responses [99–101]. Accordingly, these results should be interpreted as indicative rather than definitive evidence of immunogenicity. In addition, comprehensive in vitro and in vivo safety evaluations, such as cytokine profiling and dose optimization studies, will be essential to validate immune balance and to assess the risk of immune dysregulation before further translational development. Third, structural interaction analyses provide insights into the potential compatibility between the vaccine construct and innate immune receptors, but do not reflect dynamic signaling processes or functional receptor activation. Therefore, comprehensive in vitro and in vivo immunological studies will be required to validate the predicted immune responses, assess functional efficacy, and determine the translational potential of the vaccine construct.

## Conclusions

Our studies demonstrated the efficacy of multiple-epitope vaccine derived from neoantigens generated by hub genes, which serve as central molecular regulators in tumorigenesis. To our knowledge, this represents the first systematic effort to integrate hub genes for epitope prediction for LUAD immunotherapy. In silico assessments showed that the MEV is antigenic, immunogenic, stable and non-toxic. Despite promising in silico predictions, the anti-tumor efficacy of the MEV construct requires rigorous experimental validation through: (1) murine tumor challenge models to assess tumor regression rates; (2) flow cytometric quantification of CD8 + T-cell infiltration; and (3) longitudinal monitoring of immune memory formation.

## Supplementary Information

Supplementary Material 1

Supplementary Material 2

## Data Availability

All relevant data used in this study are included in the manuscript and supplementary files.

## Abbreviations

LUAD: Lung adenocarcinoma
NSCLC: Non-small cell lung cancer
MEV: Multi-epitope vaccine
TSAs: Tumor-specific antigens
CTL: Cytotoxic T-lymphocyte
ICIs: Immune checkpoint inhibitors
MAGE-A3: Melanoma antigen family A
NY-ESO-1: New York esophageal squamous cell carcinoma 1
TLR3: Toll-like receptor 3
GEO: Gene Expression Omnibus
DEGs: Differentially expressed genes
PPIs: Protein-protein interactions
MCC: Maximal Clique Centrality
MNC: Maximum Neighborhood Component
HRs: Hazard ratios
SNV: Single nucleotide variants
PADRE: The pan HLA DR-binding epitope
His-tag: Poly-histidine tag
PI: Theoretical isoelectric point
GDT-HA: Global distance test-high accuracy
RMSD: Root-mean-square deviation
NMA: Normal mode analysis
CAI: Codon adaptation index
GRAVY: Grand Average of Hydropathicity
DC: Dendritic cell
TIL: Tumor infiltrating lymphocyte
