# Supplementary material for: In silico design of the multi-epitope vaccine for lung adenocarcinoma based on hub gene-derived neoantigens
Source: BMC Cancer. 2026 Mar 6;26:476. doi: 10.1186/s12885-026-15765-1 (PMC13077948; doi:10.1186/s12885-026-15765-1)
Supplement: Supplementary file 1 — Supplementary Material 1 [file 12885_2026_15765_MOESM1_ESM.pdf]

# Supplementary data

After merging Figures B and C, the result of A and Figure 8B containing the corresponding molecular weight was obtained; As shown in Figure8B, the molecular weight of MEV is appoximiatly 25 kDa, which is consistent with the expected molecular weight.

Figure 8B

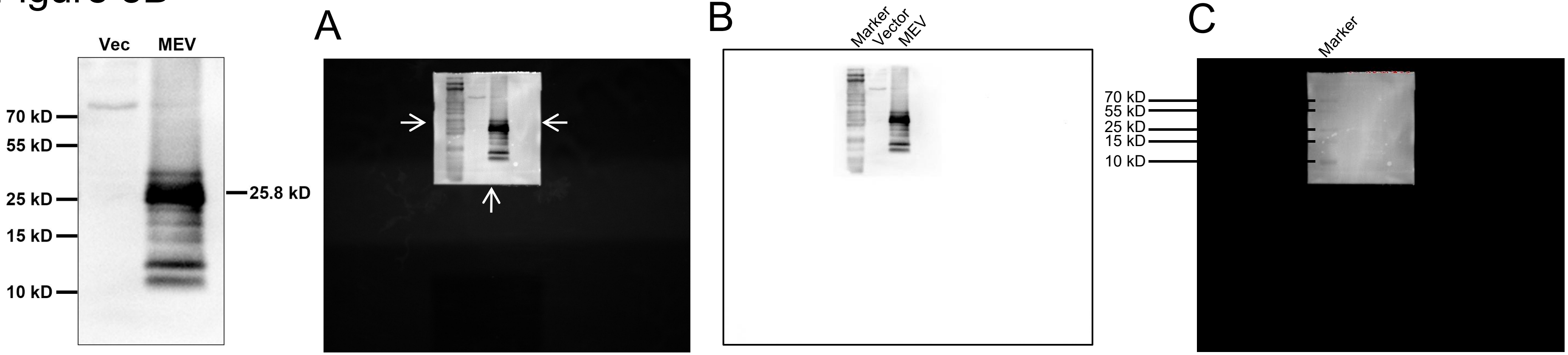


**Figure 8B and its original data.** (A) Fused exposure and white-light image. (B) Original Western blot exposure image for  Figure 8B; (C) Original Western blot white-light image for  Figure 8B. The white arrows indicate the edge of the membrane.

## Repeat data of Figure 8B

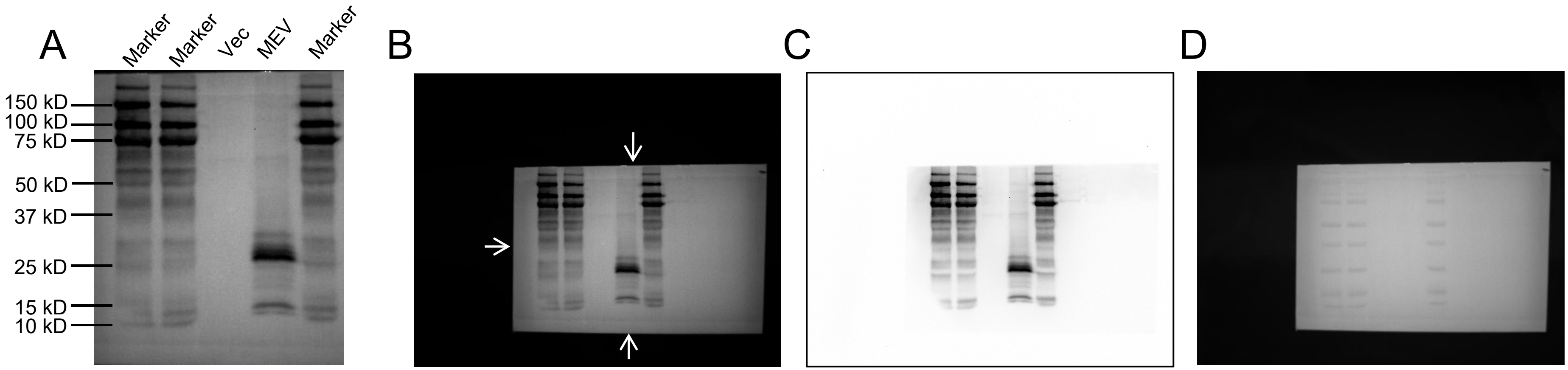


**Repeat data of Figure 8B.**(A) Western Blot with molecular weight markers indicated; (B) Fused exposure and white-light image. (C) Original Western blot exposure image; (D) Original Western blot white light image. The white arrows indicate the edge of the membrane.
