# Supplementary material for: In silico design of the multi-epitope vaccine for lung adenocarcinoma based on hub gene-derived neoantigens
Source: BMC Cancer. 2026 Mar 6;26:476. doi: 10.1186/s12885-026-15765-1 (PMC13077948; doi:10.1186/s12885-026-15765-1)
Supplement: Supplementary file 2 — Supplementary Material 2 [file 12885_2026_15765_MOESM2_ESM.zip › Additional file 1/New supplementary.pptx]

## Slide 1
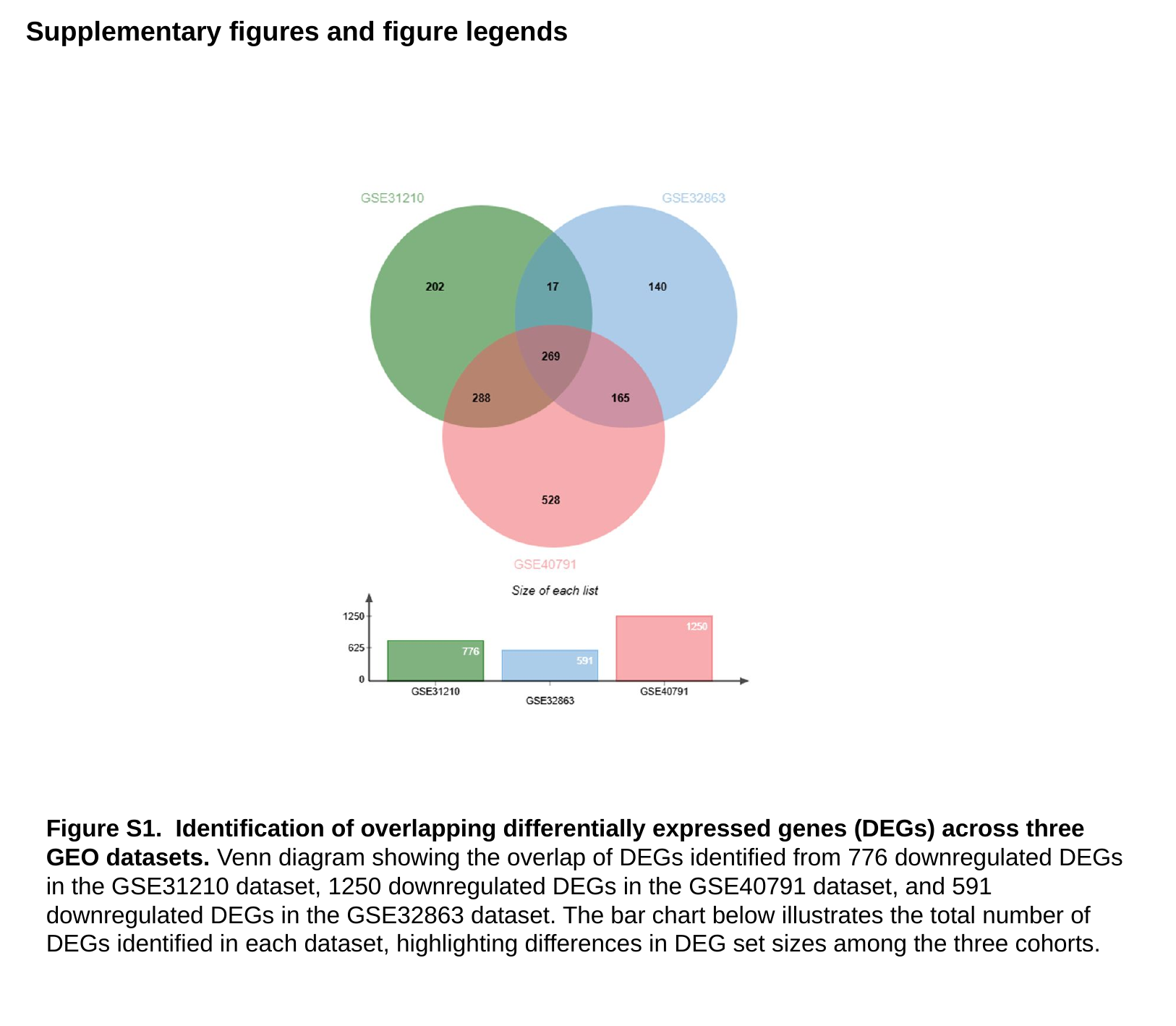

Supplementary figures and figure legends
Figure S1. Identification of overlapping differentially expressed genes (DEGs) across three GEO datasets. Venn diagram showing the overlap of DEGs identified from 776 downregulated DEGs in the GSE31210 dataset, 1250 downregulated DEGs in the GSE40791 dataset, and 591 downregulated DEGs in the GSE32863 dataset. The bar chart below illustrates the total number of DEGs identified in each dataset, highlighting differences in DEG set sizes among the three cohorts.

## Slide 2
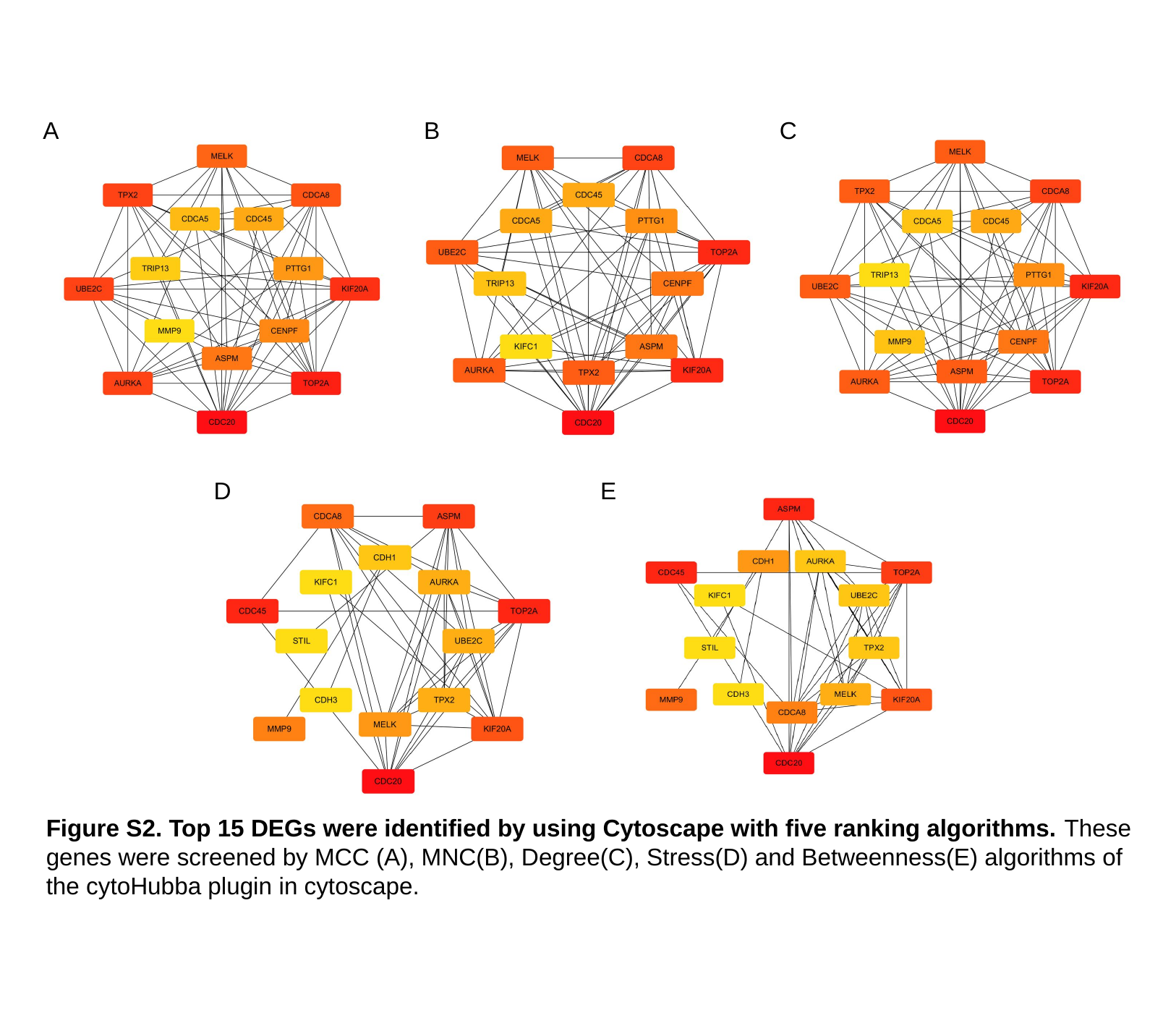

A
B
C
D
E
Figure S2. Top 15 DEGs were identified by using Cytoscape with five ranking algorithms. These genes were screened by MCC (A), MNC(B), Degree(C), Stress(D) and Betweenness(E) algorithms of the cytoHubba plugin in cytoscape.

## Slide 3
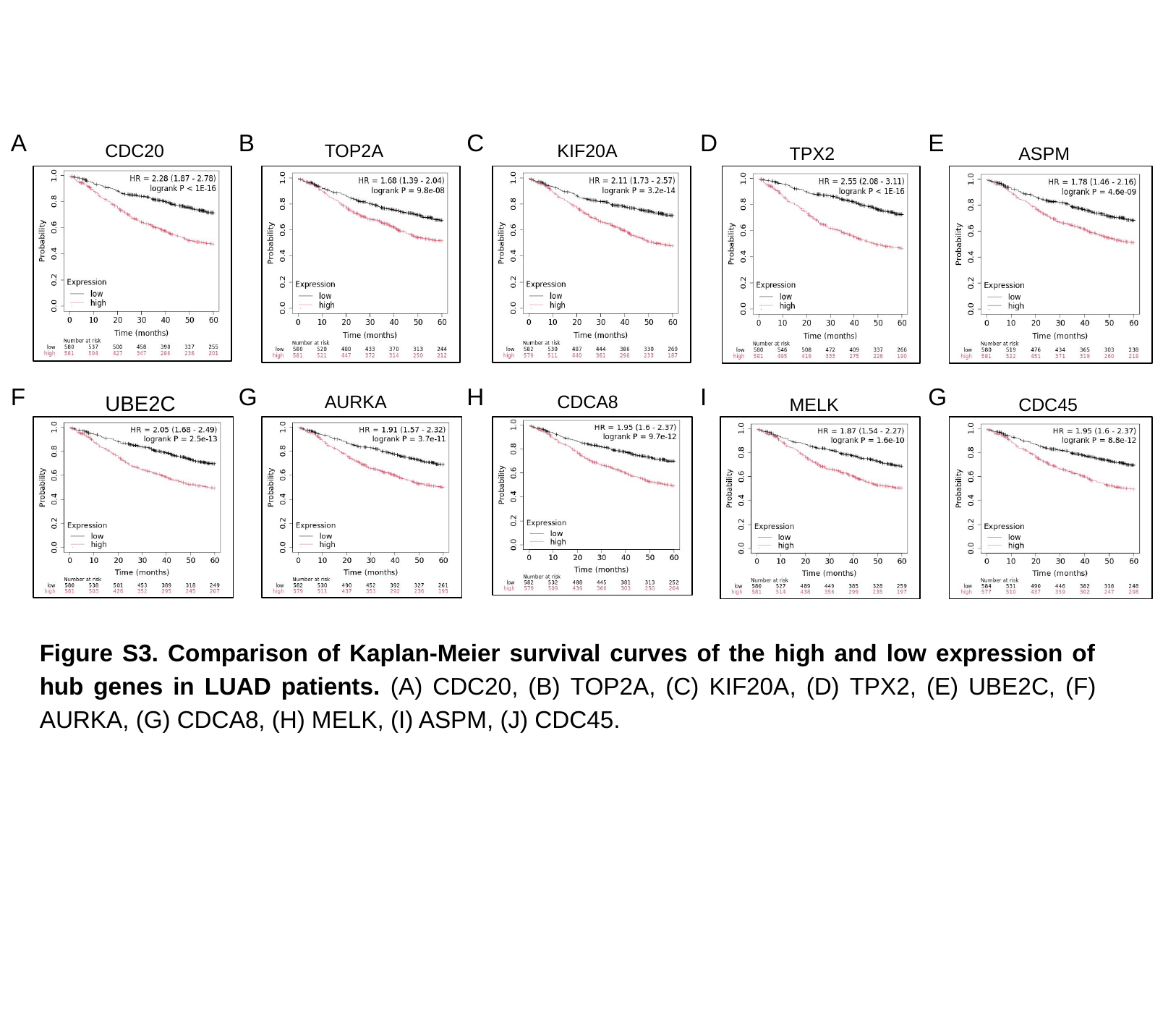

A
B
C
D
E
CDC20
TOP2A
KIF20A
TPX2
ASPM
F
G
H
I
G
UBE2C
AURKA
CDCA8
MELK
CDC45
Figure S3. Comparison of Kaplan-Meier survival curves of the high and low expression of hub genes in LUAD patients. (A) CDC20, (B) TOP2A, (C) KIF20A, (D) TPX2, (E) UBE2C, (F) AURKA, (G) CDCA8, (H) MELK, (I) ASPM, (J) CDC45.

## Slide 4
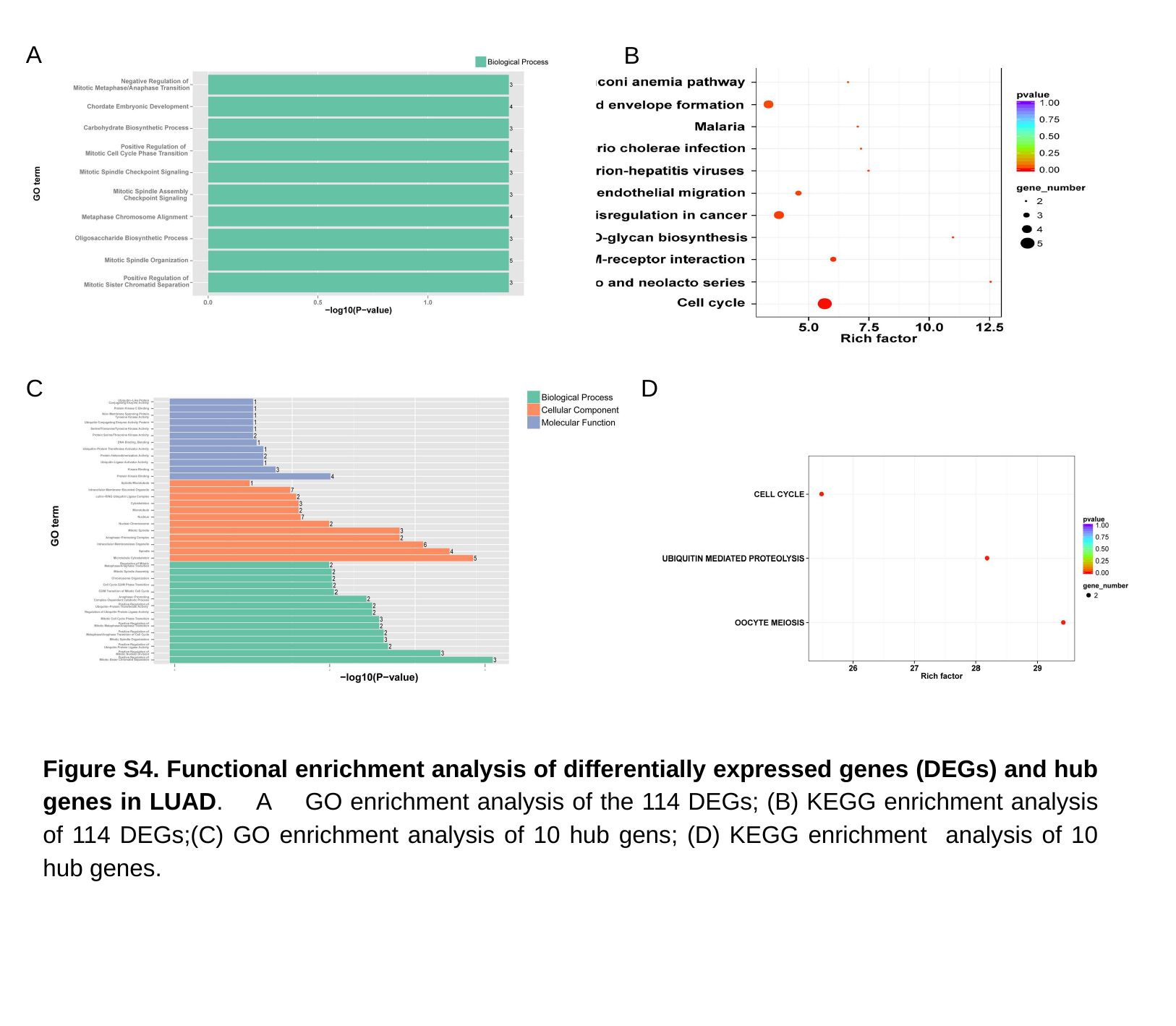

A
B
C
D
Figure S4. Functional enrichment analysis of differentially expressed genes (DEGs) and hub genes in LUAD.（A）GO enrichment analysis of the 114 DEGs; (B) KEGG enrichment analysis of 114 DEGs;(C) GO enrichment analysis of 10 hub gens; (D) KEGG enrichment analysis of 10 hub genes.

## Slide 5
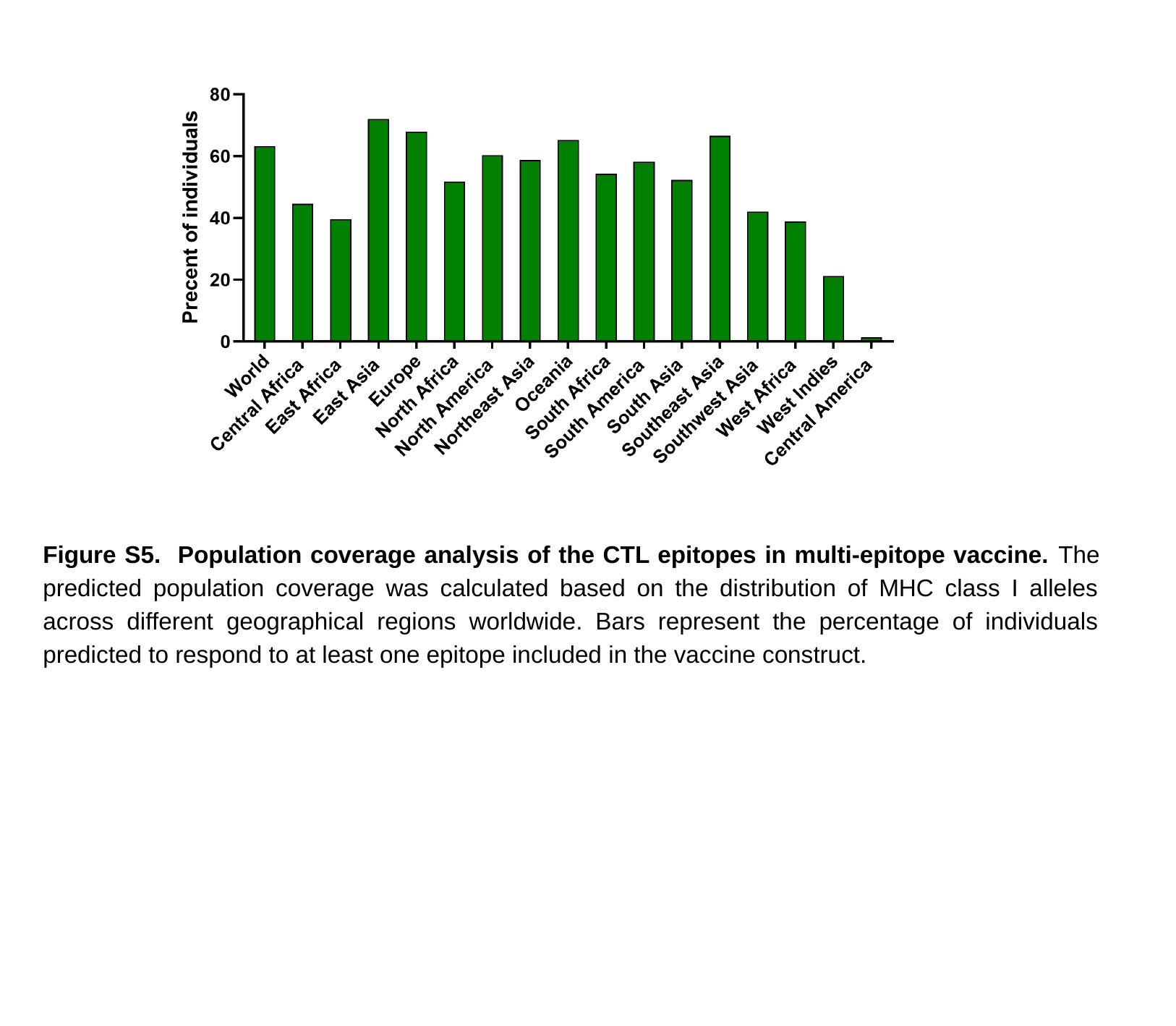

Figure S5. Population coverage analysis of the CTL epitopes in multi-epitope vaccine. The predicted population coverage was calculated based on the distribution of MHC class I alleles across different geographical regions worldwide. Bars represent the percentage of individuals predicted to respond to at least one epitope included in the vaccine construct.

## Slide 6
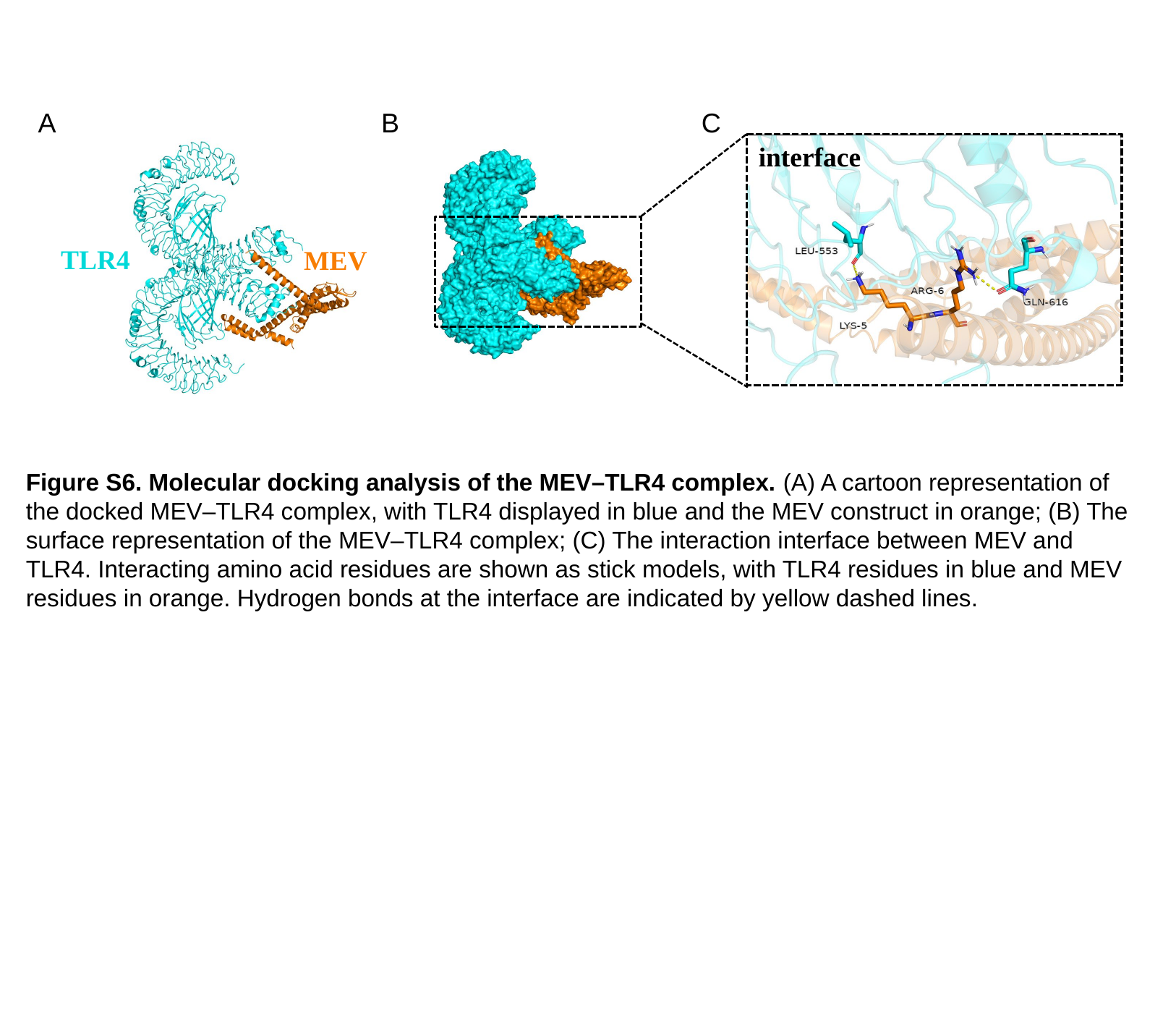

A
B
C
interface
TLR4
MEV
Figure S6. Molecular docking analysis of the MEV–TLR4 complex. (A) A cartoon representation of the docked MEV–TLR4 complex, with TLR4 displayed in blue and the MEV construct in orange; (B) The surface representation of the MEV–TLR4 complex; (C) The interaction interface between MEV and TLR4. Interacting amino acid residues are shown as stick models, with TLR4 residues in blue and MEV residues in orange. Hydrogen bonds at the interface are indicated by yellow dashed lines.
