# Supplementary material for: In silico design of the multi-epitope vaccine for lung adenocarcinoma based on hub gene-derived neoantigens
Source: BMC Cancer. 2026 Mar 6;26:476. doi: 10.1186/s12885-026-15765-1 (PMC13077948; doi:10.1186/s12885-026-15765-1)
Supplement: Supplementary file 2 — Supplementary Material 2 [file 12885_2026_15765_MOESM2_ESM.zip › Additional file 1/Supplementary Table 3.docx]

**Supplementary Table 3. Key interacting residue pairs between the MEV vaccine construct and different TLR subtypes identified by molecular docking analysis.**

| **Receptor type** | **Receptor** | **Vaccine** | **Type of bond** | **Distance(Å)** |
| --- | --- | --- | --- | --- |
| TLR2 | GLH 217 | SER 23 | Hydrogen bond | 2.9 |
|  | HIS 193 | SER 23 | Hydrogen bond | 2.6 |
|  | HIE 78 | LEU 13 | Hydrogen bond | 2.7 |
|  | TYR 275 | TYR 37 | π-π interaction | 5.7 |
| TLR3 | HIS 359 | HIS 134 | Hydrogen bond | 2.5 |
|  | HIS 156 | ARG 160 | Hydrogen bond | 2.0 |
|  | GLN 62 | HIE 229 | Hydrogen bond | 2.8 |
|  | LYS 41 | HIE 229 | Hydrogen bond | 2.6 3.4 |
|  | ASP 280 | HIP 146 | salt bridge | 3.8 |
|  | LYS 41 | HIE 229 | salt bridge | 4.2 |
|  | TYR 326 | HIS 134 | π-π interaction | 3.6 |
|  | TYR 326 | TYR 142 | π-π interaction | 4.3 |
| TLR4 | GLN 616 | ARG 6 | Hydrogen bond | 3.0 |
|  | LEU 553 | LYS 5 | Hydrogen bond | 3.1 |

Note: HIE and HIP denote different protonation states of histidine, whereas GLH represents the protonated form of glutamate.
