# Supplementary material for: In silico design of the multi-epitope vaccine for lung adenocarcinoma based on hub gene-derived neoantigens
Source: BMC Cancer. 2026 Mar 6;26:476. doi: 10.1186/s12885-026-15765-1 (PMC13077948; doi:10.1186/s12885-026-15765-1)
Supplement: Supplementary file 2 — Supplementary Material 2 [file 12885_2026_15765_MOESM2_ESM.zip › Additional file 1/Supplementary Table 4.docx]

**Supplementary Table 4. MM-GBSA binding free energies (kcal/mol) and individual energy components of MEV complexes with TLR2 and TLR3.**

| **Binding free energy（kcal/mol）** | **TLR2** | **TLR3** |
| --- | --- | --- |
| MMGBSA_ΔG_Bind | -122.2397839 | -99.71599751 |
| MMGBSA_ΔG_Bind_Coulomb | 121.206839233142 | -462.6850288 |
| MMGBSA_ΔG_Bind_Covalent | -5.404201243 | 5.14465602766177 |
| MMGBSA_ΔG_Bind_Hbond | -11.63759048 | -10.46948633 |
| MMGBSA_ΔG_Bind_Lipo | -37.63018531 | -31.91017994 |
| MMGBSA_ΔG_Bind_Packing | -2.00735735 | -9.660069319 |
| MMGBSA_ΔG_Bind_SelfCont | 2.42493041105291 | -0.143156161 |
| MMGBSA_ΔG_Bind_SolvGB | -49.02095773 | 561.779117570108 |
| MMGBSA_ΔG_Bind_vdW | -140.1712614 | -151.7718506 |
